# TripletGO: Integrating Transcript Expression Profiles with Protein Homology Inferences for Gene Function Prediction

**DOI:** 10.1016/j.gpb.2022.03.001

**Published:** 2022-05-11

**Authors:** Yi-Heng Zhu, Chengxin Zhang, Yan Liu, Gilbert S. Omenn, Peter L. Freddolino, Dong-Jun Yu, Yang Zhang

**Affiliations:** 1 School of Computer Science and Engineering, Nanjing University of Science and Technology, Nanjing 210094, China; 2 Department of Computational Medicine and Bioinformatics, University of Michigan, Ann Arbor, MI 48109, USA; 3 Departments of Internal Medicine and Human Genetics, and School of Public Health, University of Michigan, Ann Arbor, MI 48109, USA; 4 Department of Biological Chemistry, University of Michigan, Ann Arbor, MI 48109, USA

**Keywords:** Gene function annotation, Gene Ontology, Transcript expression profile, Triplet network, Protein-level alignment

## Abstract

**Gene Ontology** (GO) has been widely used to annotate functions of genes and gene products. Here, we proposed a new method, TripletGO, to deduce GO terms of protein-coding and non-coding genes, through the integration of four complementary pipelines built on **transcript expression profile**, genetic sequence alignment, protein sequence alignment, and naïve probability. TripletGO was tested on a large set of 5754 genes from 8 species (human, mouse, *Arabidopsis*, rat, fly, budding yeast, fission yeast, and nematoda) and 2433 proteins with available expression data from the third Critical Assessment of Protein Function Annotation challenge (CAFA3). Experimental results show that TripletGO achieves function annotation accuracy significantly beyond the current state-of-the-art approaches. Detailed analyses show that the major advantage of TripletGO lies in the coupling of a new **triplet network**-based profiling method with the feature space mapping technique, which can accurately recognize function patterns from transcript expression profiles. Meanwhile, the combination of multiple complementary models, especially those from transcript expression and **protein-level alignments**, improves the coverage and accuracy of the final GO annotation results. The standalone package and an online server of TripletGO are freely available at https://zhanggroup.org/TripletGO/.

## Introduction

In the post-genome sequencing era, a major challenge is to annotate the biological functions of all genes and gene products, which are grouped, in the context of the widely used Gene Ontology (GO), into three aspects, *i.e.*, molecular function (MF), biological process (BP), and cellular component (CC) [1]. Accurate annotation of gene functions provides essential knowledge to disease mechanisms and drug design [2], [3]. Direct determination of the functions of genes via biochemical or genetic experiments is typically time-consuming and laborious, and often incomplete [4]. As a result, a large number of genes in the sequenced genomes have no available function annotation to date. For example, according to official statistics in the neXtProt platform [5], nearly 2000 human protein-coding genes have yet no known functions; for many other organisms of biomedical or industrial importance, annotation rates are substantially lower. To fill the gap between sequence and function, it is urgent to develop efficient computational algorithms for function prediction [6], [7].

Function annotations can be performed at either the protein or gene level. In the former case, the function of the query gene is determined by that of its encoded protein, which can be deduced from the protein sequence, structure, or family information [8], [9], [10], [11], [12], [13], [14]. However, protein-coding genes account for only ∼ 2% of a typical multicellular eukaryote genome such as that of humans [15]. There are also many genes for non-coding RNAs as well as genes whose coding potential is unknown or ambiguous.

Most gene-level annotation methods deduce GO terms for queries by using a guilt-by-association (GBA) strategy, which is typically based on the similarity of expression profiles between the gene of interest and template genes with known GO annotations [16], [17], [18]. The rationale of GBA is reasonable as genes with the same functions often show similar expression profiles. This was supported by the third Critical Assessment of Protein Function Annotation challenge (CAFA3), which showed that expression profile has a great potential to improve prediction performance [19]. Despite that there are some achievements of current expression profile-based methods, challenges remain.

First, it is tricky to define an effective similarity measure of expression profiles as the substitute for functional similarity. In previous work, several unsupervised methods (*e.g.*, Pearson correlation coefficient [20] and mutual rank [21]) and supervised methods (*e.g.*, metric learning for co-expression [17]) have been developed to measure the expression profile similarity in gene function prediction. Unfortunately, these methods cannot achieve optimal performance, because these expression similarity metrics may have no close correlation with functional similarity. Part of the reason is that these methods define the expression similarity in the original space, in which the expression data show a high dimensionality across multiple tissues and complicated distributions; as a result, the measured expression similarity is hardly associated with functional similarity and thus a higher expression similarity (by these metrics) often does not indicate a higher functional similarity. To address this issue, a promising approach is to change the data distribution via feature space mapping [22], in which the expression profiles are mapped from the original feature space to a new embedding space by non-linear functions, and the expression similarity is then associated with functional similarity in this embedding space. The second challenge is that the functional similarity of genes is often difficult to be completely captured by one similarity measure. This necessitates the combinations of multiple similarity measures from different biological datasets which may help improve both accuracy and coverage of function prediction [9].

In this work, we proposed and tested a new approach, TripletGO, to integrate multi-source information from both genes and proteins for protein-coding and non-coding gene annotations. First, we extended a supervised triplet network method [23] to assess expression profile similarities in function prediction. In this extended triplet network pipeline (TNP), the expression profiles are mapped from the original feature space to an embedding feature space via deep neural network learning, where a triplet loss function is designed to enhance the correlation between expression profile and gene function. Second, considering that most protein-coding gene functions are performed through proteins and that protein sequence alignments, which are based on 20 amino acids, often provide more specific function associations than nucleotide sequence alignments, we proposed a protein-level method for GO prediction using protein sequence similarity. Finally, a composite model was derived by integrating the output of four complementary GO prediction pipelines, built on the TNP-based expression profile, genetic sequence alignment, naïve probability, and protein sequence alignment, through an optimal neural network training. TripletGO has been systematically tested on a large set of non-redundant genes collected from eight species, where the results demonstrated the significant advantage of TripletGO on accurate GO term prediction over the current state-of-the-art approaches in the field. The standalone package and an online server of TripletGO are freely available at https://zhanggroup.org/TripletGO/.

## Method

### Overview of TripletGO

**Figure 1:**
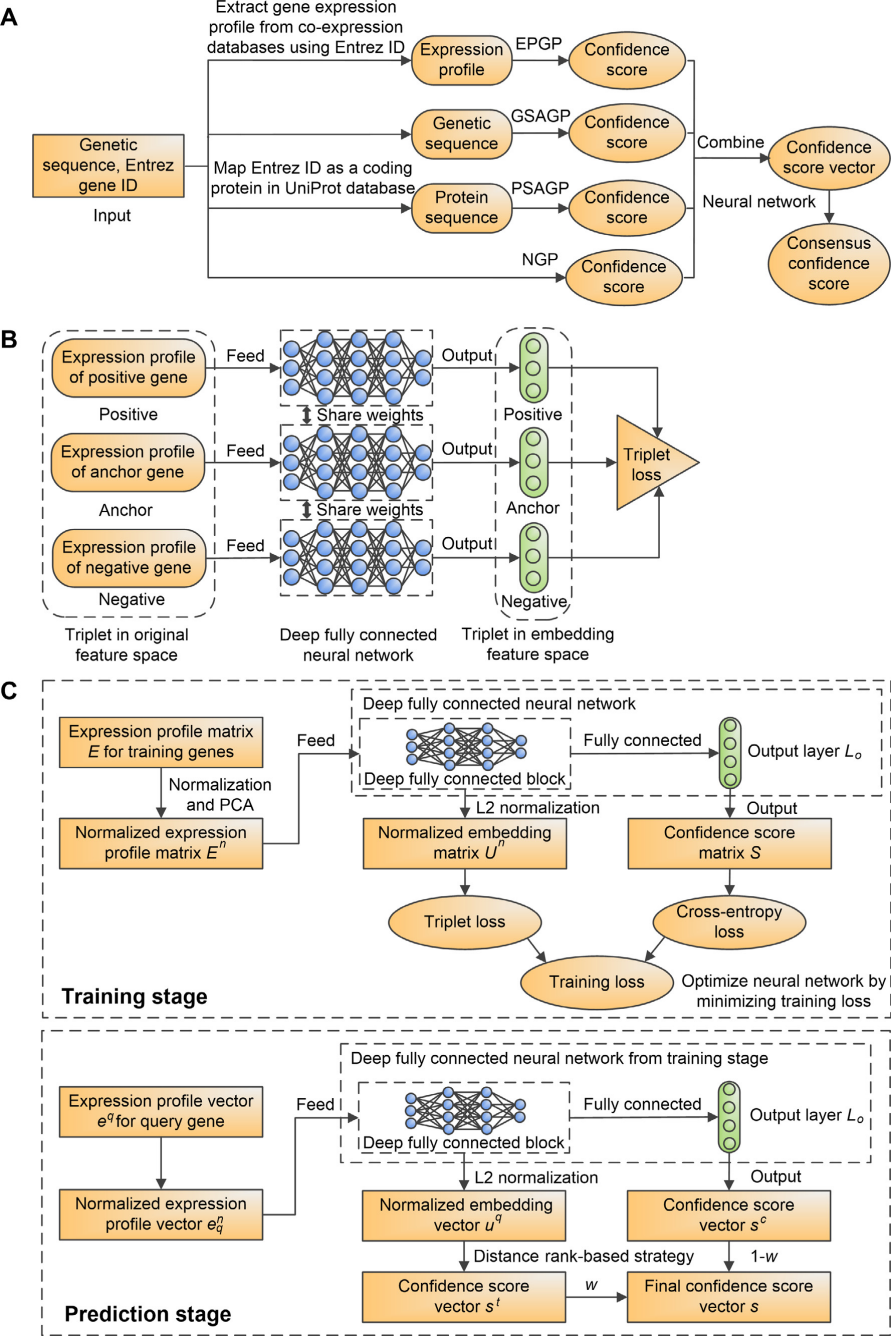
**The procedureof TripletGO** **A.** The flowchart of TripletGO to integrate four complementary pipelines for GO prediction. **B.** The design of a triplet network for assessing expression profile similarity. **C.** TNP for EPGP. GO, Gene Ontology; EPGP, expression profile-based GO prediction; GSAGP, genetic sequence alignment-based GO prediction; PSAGP, protein sequence alignment-based GO prediction; NGP, naïve-based GO prediction; ID, identity document; TNP, triplet network pipeline.

TripletGO is a hierarchical approach for gene function annotation with respect to GO terms, as shown in Figure 1A. In TripletGO, the final GO model is a combination of the outputs of four complementary pipelines, including expression profile-based GO prediction (EPGP) by TNP, genetic sequence alignment-based GO prediction (GSAGP), protein sequence alignment-based GO prediction (PSAGP), and naïve-based GO prediction (NGP). Here, the input is a genetic sequence with Entrez gene ID, and the output is the confidence score for the predicted GO term. First, we extract the expression profile and coding protein sequence for a query gene from COXPRESdb [24] (or ATTED-II [21]) database and UniProt [25] database, respectively, using Entrez ID. Then, the expression profile, genetic sequence, and protein sequence are respectively used as the inputs of EPGP, GSAGP, and PSAGP methods to output the confidence scores of GO terms. Moreover, NGP method is also used to calculate the confidence score. Finally, for a GO term $Q_{i}$, its confidence scores by the four methods are serially combined as a vector, which is used as the input of a fully connected neural network to output the consensus score.

### EPGP by TNP

In EPGP, a triplet network [23] is used to measure the similarity of expression profiles, as shown in Figure 1B. The input is a triplet variable $\left(anc,\;pos,neg\right)$, where anc is an anchor (baseline) gene, pos is a positive gene with the same function as anc, and neg is a negative gene with a different function from anc. First, the expression profile of each gene is mapped from the original feature space to an embedding space using the same deep neural network. Next, the expression dissimilarity between two genes in embedding space is measured by Euclidean distance (ED) [26] of the mapped expression profiles. Finally, the triplet loss function is designed to associate expression similarity with functional similarity:

(1) $$\mathrm{Tripletloss}=max(d\left(anc,\;pos\right)+margin-d\left(anc,\;neg\right),\;0)\;$$

where $d\left(anc,\;pos\right)$ is the ED between anchor and positive genes in embedding space, $d\left(anc,\;neg\right)$ is the distance between anchor and negative genes, and margin is a pre-set positive value. Here, the minimization of the triplet loss requests for the maximization of $d\left(anc,\;neg\right)-d\left(anc,\;pos\right)$. In the ideal case, Tripletloss=0 when $d\left(anc,\;neg\right)\geq d\left(anc,\;pos\right)+margin$, which indicates substantially higher similarity (lower distance) of the anchor gene to the positive gene than to the negative gene.

It has been demonstrated that cross-entropy loss [27] helps to improve the performance of triplet network [28], [29]. Therefore, we further combine the triplet loss with the cross-entropy loss in the TNP to predict gene function from expression profiles. The overall workflow of TNP is depicted in Figure 1C, which contains two stages.

### Training stage of TNP

#### Procedure I: expression profile normalization

In a training dataset, the expression profiles of all m genes are represented as a matrix $E={\left(e_{\mathrm{ij}}\right)}_{m\times l}$, where l is the number of experimental samples in microarray technology [30], and $e_{\mathrm{ij}}$ is the expression value of the i-th gene on the j-th sample. Each row of E can be viewed as the expression profile of a gene. To reduce noise and computing cost, the matrix E is transformed into a normalized matrix $E^{n}={\left(e_{\mathrm{ij}}^{n}\right)}_{m\times h}$ (h<l) by performing z-score normalization [31] and principal component analysis (PCA) [32].

#### Procedure II: expression profile mapping using a neural network

The normalized expression profiles are mapped from the original feature space to an embedding space using a neural network. Specifically, the normalized matrix $E^{n}$ is fed to a deep fully connected block (DFCB) with N layers to output an embedding matrix $U={\left(u_{\mathrm{ij}}\right)}_{m\times d_{N}}$, where $d_{N}$ is number of neurons in the N-th layer. Then, L2 normaliztion is executed on U to obtain a normalized matrix $U^{n}={\left(u_{\mathrm{ij}}^{n}\right)}_{m\times d_{N}}$, where $u_{\mathrm{ij}}^{n}=u_{\mathrm{ij}}/{\left(\sum_{j=1}^{d_{N}}{u_{\mathrm{ij}}}^{2}\right)}^{1/2}$. Each row of $U^{n}$ can be viewed as the expression profile of a gene in the embedding space.

At the same time, an output layer $L_{O}$ with sigmoid activation function [33] is fully connected with DFCB to output a score matrix $S={\left(s_{\mathrm{ij}}\right)}_{m\times r}$, where r is the number of GO terms in the training dataset, and $s_{\mathrm{ij}}$ is the confidence score that the i-th training gene is associated with the j-th GO term. Then, we calculate triplet loss and cross-entropy loss based on matrix $U^{n}$ and score matrix S, respectively.

#### Procedure III: loss function calculation and network optimization

We use the “batch on hard” strategy [34], [35] to calculate the triplet loss:

(2) $${\mathrm{Tripletloss}}_{\mathrm{hard}}=\sum_{i=1}^{m}\max\left({d\left(i,\;pos\right)}_{\max}+margin-{d\left(i,\;neg\right)}_{\min},0\right)/m$$

where ${d\left(i,\;pos\right)}_{\max}$ (or ${d\left(i,\;neg\right)}_{\min}$) is the maximum (or minimum) value of distances between the i-th gene and all positive (or negative) genes with same (or different) function of the i-th gene in embedding space. The distance between the two genes $\left(i,j\right)$ is measured by $d\left(i,j\right)=\sum_{k=\;1}^{d_{N}}{\left(u_{\mathrm{ik}}^{n}-u_{\mathrm{jk}}^{n}\right)}^{2}/4$, where the division factor of 4 is introduced to normalize d(i,j) into the range of [0,1], *i.e.*, $0\leq d\left(i,j\right)\leq \sum_{k=\;1}^{d_{N}}\left(2{{(u}_{\mathrm{ik}}^{n})}^{2}+{{2(u}_{\mathrm{jk}}^{n})}^{2}\right)/4=1$. Moreover, two genes are considered to have the same function if their functional similarity is larger than a cut-off value $c_{f}$. The functional similarity of two genes is measured by the F1-score between their GO terms, as shown in File S1A.

The cross-entropy loss is calculated as:

(3) $${\mathrm{Loss}}_{\mathrm{cross}-entropy}=-\overset{m}{\underset{i=1}{\sum }}\left(\overset{r}{\underset{j=1}{\sum }}y_{\mathrm{ij}}∙\log\left(s_{\mathrm{ij}}\right)+\left(1-y_{\mathrm{ij}}\right)log\left(1-s_{\mathrm{ij}}\right)\right)/\left(r∙m\right)$$

where $y_{\mathrm{ij}}=1$ if the i-th gene is associated with the j-th GO term in the experimental function annotation; otherwise, $y_{\mathrm{ij}}=0$.

The final training loss in TNP is the combination of triplet loss and cross-entropy loss:

(4) $$\mathrm{Trainingloss}={\mathrm{Triletloss}}_{\mathrm{hard}}+\alpha ∙{\mathrm{Loss}}_{\mathrm{cross}-entropy}$$

where α is a trade-off value. Finally, we minimize training loss to optimize neural network by Adam optimization algorithm [36].

### Prediction stage of TNP

The input is a query gene with expression profile vector $e^{q}$, and the output is a confidence score vector s, including the confidence scores of r GO terms for query. First, z-score normalization and PCA are orderly executed on $e^{q}$ to obtain a normalized vector $e_{q}^{n}$, which was used as the input of DFCB. Then, we execute L2 normalization on the output of DFCB to obtain a normalized embedding vector $u^{q}$. Next, a distance rank-based strategy (see details in File S1B) is executed on the normalized embedding matrix of training genes ($U^{n}$) and $u^{q}$ to generate a confidence score vector $s^{t}$. At the same time, the output layer $L_{O}$ outputs another score vector $s^{c}$ by sigmoid function mapping. The final score vector s is the combination of two vectors:

(5) $$s=w∙s^{t}+\left(1-w\right)∙s^{c}$$

where w is a trade-off value and ranges from 0 to 1.

### GSAGP

In GSAGP, we search the template genes, which have the similar sequences with query gene, from a genetic sequence database with GO annotation (named GSD-GOA, see the “Datasets” section below) for functional annotation.

For a query, we extract its RNA sequence from National Center for Biotechnology Information (NCBI) [37]. Then, Blastn [38] is used to search the templates of query with an e-value cutoff of 0.1 against GSD-GOA. To remove homology contamination, we exclude all homologous templates which have more than $t_{1}$ sequence identity with the query. Finally, the remaining templates are used to annotate the query. Specifically, the confidence score that the query is associated with GO term $Q_{i}$ is calculated as:

(6) $${S\left(Q_{i}\right)}_{\mathrm{GSAGP}}=\frac{\sum_{k=1}^{n}b_{k}∙I_{k}\left(Q_{i}\right)}{\sum_{k=1}^{n}b_{k}}$$

where n is the number of template genes, $b_{k}$ is the bit-score of k-th template by Blastn; $I_{k}\left(Q_{i}\right)=1$, if the k-th template is associated with $Q_{i}$ in the experimental function annotation; otherwise, $I_{k}\left(Q_{i}\right)=0$.

### PSAGP

In PSAGP, we select the template genes, whose coding proteins have similar sequence with that of the query, for GO functional annotation.

For a query gene, we map it as the corresponding coding protein sequence *P* in the UniProt database [25]. Then, Blastp [38] is used to search the template proteins of *P* with a e-value cutoff of 0.1 against a protein sequence database (*i.e.*, PSD, see the “Datasets” section below), where homologous templates with a sequence identity above $t_{2}$ to *P* are removed. Finally, the remaining templates are mapped back to the genes in a gene-level GO annotation database (named Gene-GOA, see the “Datasets” section below) to annotate the query. The confidence score is calculated using the same scoring function as in GSAGP [*i.e.*, Equation (6)], where $b_{k}$ is the bit-score of the k-th template by Blastp.

### NGP

In NGP, the confidence score that a query is associated with GO term $Q_{i}$ can be calculated by the frequency of $Q_{i}$ in Gene-GOA:

(7) $${S\left(Q_{i}\right)}_{\mathrm{NGP}}=N\left(Q_{i}\right)/N_{\mathrm{GO}}$$

where $N(Q_{i})$ is the number of genes associated with $Q_{i}$, and $N_{\mathrm{GO}}$ is the number of genes with at least one annotation for the same GO aspect as $Q_{i}$. This predictor can be thought of as a prior arising from the overall abundance of a particular annotation in Gene-GOA.

### Consideration of hierarchical relation for evaluation of GO annotation

The GO annotation is hierarchical [19]. Specifically, for both the ground truth and the prediction, if a protein (gene) is annotated with a GO term $Q_{i}$, it should be annotated with the direct parent and all ancestors of $Q_{i}$. To enforce this hierarchical relation, we follow CAFA’s rule and use a common post-processing procedure [10] for the confidence score of term $Q_{i}$ in all GO prediction methods as follows:

(8) $${S\left(Q_{i}\right)}_{\mathrm{post}}=max\;\left(S\left(Q_{i}\right),\;{S\left({\mathrm{QC}}_{i}^{1}\right)}_{\mathrm{post}},{S\left({\mathrm{QC}}_{i}^{2}\right)}_{\mathrm{post}},\;\cdots ,\;{S\left({\mathrm{QC}}_{i}^{n}\right)}_{\mathrm{post}}\right)$$

where $S\left(Q_{i}\right)$ and ${S\left(Q_{i}\right)}_{\mathrm{post}}$ are the confidence scores of $Q_{i}$ before and after post-processing, $S{\left({\mathrm{QC}}_{i}^{1}\right)}_{\mathrm{post}},\;S{\left({\mathrm{QC}}_{i}^{2}\right)}_{\mathrm{post}},\;\cdots ,\;\mathrm{and}\;S{\left({\mathrm{QC}}_{i}^{n}\right)}_{\mathrm{post}}$ are the confidence scores of all direct children terms of $Q_{i}$ after post-processing. This post-processing procedure enforces that the confidence score of $Q_{i}$ is larger than or equal to the scores of all children.

### Datasets

We collected all 78,170 genes with GO annotation via experimental determination from NCBI [37] to construct a gene-level GO annotation database (*i.e.*, Gene-GOA, see File S2A). The genes in Gene-GOA were used to construct the template databases (*i.e.*, GSD-GOA and PSD, see File S2B and C) in GSAGP and PSAGP, and calculate the prior probability in NGP.

To evaluate the proposed methods, we collected 57,584 genes from 8 species by the following procedures: 1) we downloaded all of 300,977 genes with expression profiles determined by microarray [30] for 20 species from COXPRESdb [24] and ATTED-II [21] databases. For each species, the total number of genes with functional annotation in Gene-GOA is shown in Table S1. Then, we selected the 8 species with the most genes with GO annotation among the 20 species, to construct benchmark datasets. 2) For each species, we randomly selected 85% of genes with GO annotation as the training dataset, and 5% genes as the validation dataset, which were separately used to construct machine learning-based models and optimize the parameters of models. The remaining 10% genes were used as the test dataset to assess the performance of models. As a result, there are 48,954, 2876, and 5754 genes in training, validation, and test datasets, respectively, for the 8 species in total, as summarized in Table S2.

### Implementation and parameter settings of TripletGO

**Supplementary figure S1:**
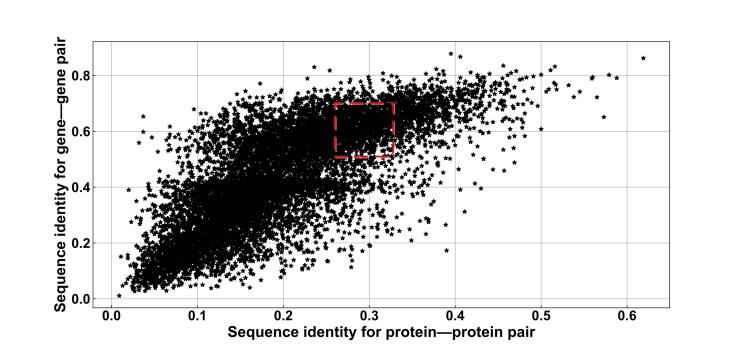
**The distribution of sequence identities for 10,000 gene–gene pairs and 10,000 mapped protein–protein pairs**.

In EPGP, DFCB consists of two fully connected layers, each including 1024 neurons with rectified linear unit (RELU) activation function [39]. The remaining parameters of EPGP are listed in Table S3. In PSAGP, we used $t_{2}$ = 30% sequence identity as the cut-off to remove homologous protein templates, following previous studies [9]. To determine the homology cutoff for nucleotide sequences, we used three different machine learning models to fit the relationship between protein sequence identity and gene sequence identity, and it was found that a 30% protein sequence identity roughly corresponds to 60% genetic sequence identity, as shown in File S3 and Figure S1. Therefore, we used $t_{1}$ = 60% sequence identity as the cut-off to remove homologous templates in the GSAGP.

### Evaluation metrices

Maximum F1-score (Fmax) and area under the precision–recall curve (AUPRC) are used to evaluate the performance of proposed methods. Fmax is one of the most important evaluation metrics in CAFA [19], [40] and is defined as:

(9) $$Fmax=\max_{0\leq t\leq 1}\left[\frac{2∙pr\left(t\right)∙rc\left(t\right)}{pr\left(t\right)+rc\left(t\right)}\right]$$

where *t* is a cut-off value of confidence score; pr(t) and rc(t) are precision and recall, respectively, with confidence score ≥t:

(10) $$\left\{\begin{array}{c} pr\left(t\right)=\frac{tp\left(t\right)}{tp\left(t\right)+fp\left(t\right)}\\ rc\left(t\right)=\frac{\mathrm{tp}(t)}{tp\left(t\right)+fn\left(t\right)} \end{array}\right)$$

where $tp\left(t\right)$ is the number of correctly predicted GO terms, $tp\left(t\right)+fp\left(t\right)$ is the number of all predicted GO terms, and $tp\left(t\right)+fn\left(t\right)$ is the number of GO terms in experimental function annotation. AUPRC is a critical measure in multi-label prediction task [41] and ranges from 0 to 1.

The average performance of a method on multiple species is measured by weighted average Fmax (WAFmax) and weighted average AUPRC (WAAUPRC):

(11) $$\left\{\begin{array}{c} WAFmax=\frac{\sum_{i=1}^{M}{Fmax}_{i}∙N_{i}}{\sum_{i=1}^{M}N_{i}}\\ WAAUPRC=\frac{\sum_{i=1}^{M}{AUPRC}_{i}∙N_{i}}{\sum_{i=1}^{M}N_{i}} \end{array}\right)$$

where M is the number of species, ${Fmax}_{i}$ and ${AUPRC}_{i}$ are the Fmax and AUPRC values, respectively, on the test dataset of the i-th species, and $N_{i}$ is the number of test genes for the i-th species.

## Results

### TNP improves EPGP

TripletGO is a hierarchical approach that takes the gene sequence, the protein sequence, and the transcript expression profile data as input. GO terms are then created by a set of four complementary pipelines, built on transcript expression profile, gene sequence alignment, protein sequence alignment, and naïve prior statistical calculation, where the final GO model is obtained by a neural network combination (Figure 1).

**Figure 2:**
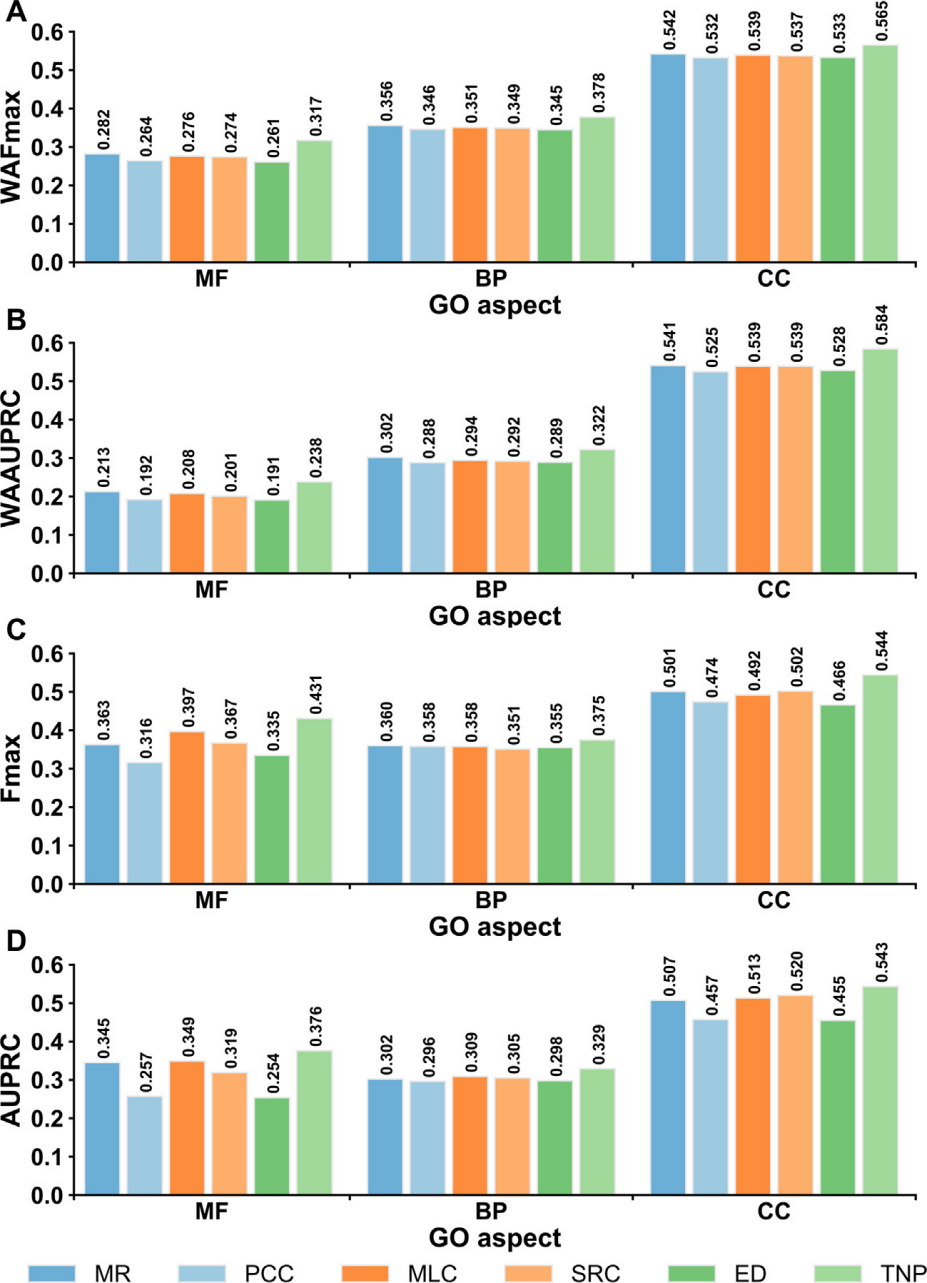
**Comparison of six transcript EPGP methods** **A.** The WAFmax values on the test datasets of 8 species. **B.** The WAAUPRC values on the test datasets of 8 species. **C.** The Fmax values on 98 non-coding test genes. **D.** The AUPRC values on 98 non-coding test genes. MR, mutual rank; PCC, Pearson correlation coefficient; MLC, metric learning for co-expression; SRC, Spearman rank correlation; ED, Euclidean distance; Fmax, maximum F1-score; AUPRC, area under the precision–recall curve; WAFmax, weighted average Fmax; WAAUPRC: weighted average AUPRC; MF, molecular function; BP, biological process; CC, cellular component.

**Supplementary figure S2:**
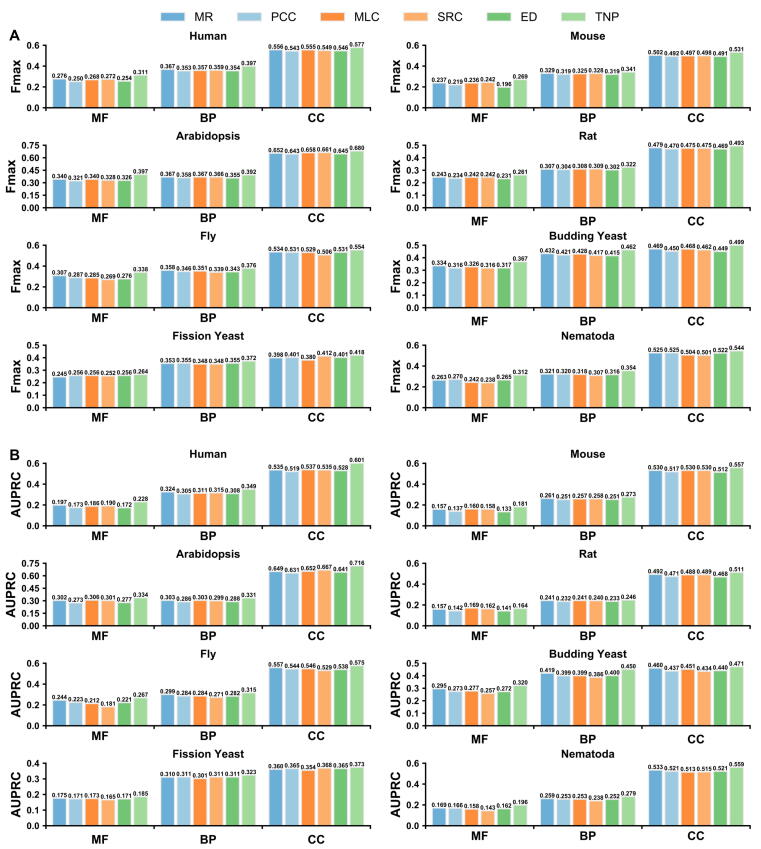
**The performance of six expression profile-based methods on the test datasets for 8 individual species**. **A**. The Fmax values of six methods for 8 species. **B**. The AUPRC values of six methods for 8 species.

**Supplementary figure S3:**
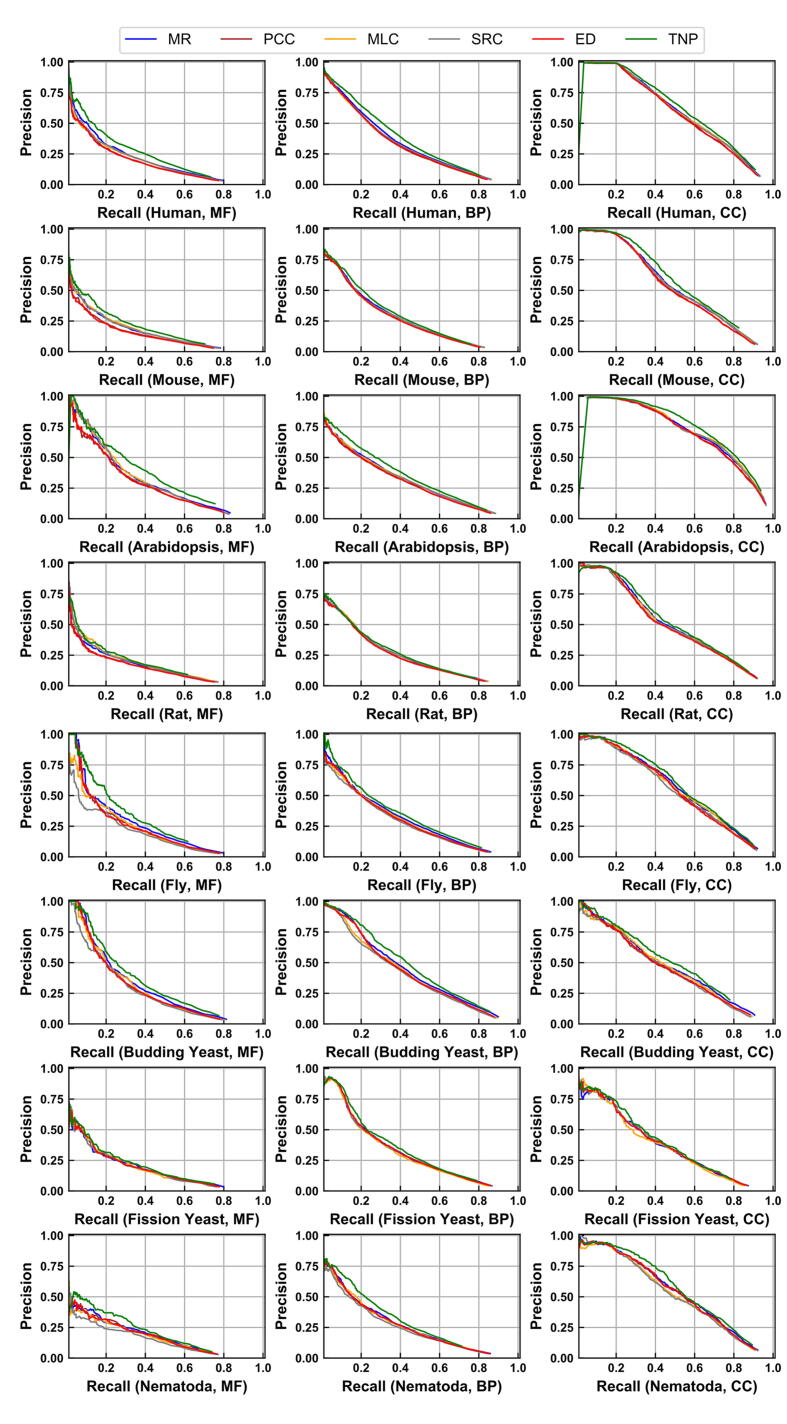
**The PRCs of six expression profile-based methods on the test datasets for 8 individual species**. PRCs, the precision-recall curves.

As TripletGO is centralized by the transcript expression profile through TNP (Figure 1C), we first compare the TNP with five existing methods in EPGP. These include four unsupervised scores: Pearson correlation coefficient (PCC) [20], Spearman rank correlation (SRC) [42], mutual rank (MR) [21], and ED [26]; and a supervised method: metric learning for co-expression (MLC) [17]. Each method is combined with the GBA strategy to predict GO terms, as described in File S4A. Figure 2A and B list the WAFmax and WAAUPRC values on the test datasets of 5754 genes from 8 species (human, mouse, *Arabidopsis*, rat, fly, budding yeast, fission yeast, and nematoda) for the six methods, where the *P* values between TNP and the other five methods in Student’s *t*-test [43] for WAFmax and WAAUPRC are summarized in Table S4. In addition, the performances of the six methods for each individual species are summarized in Figures S2 and S3 and Table S5, and discussed in File S4B.

From Figure 2A and B and Table S4, it can be observed that the accuracy of TNP is significantly higher than the other five methods in deducing function from gene expression data. Specifically, the improvements of WAFmax values between TNP and the second-best performer, MR, are 12.4% [(0.317 - 0.282)/0.282 × 100%], 6.2%, and 4.2% for MF, BP, and CC, respectively, all with *P* values significantly below 0.05. At the same time, TNP achieves an increase of WAAUPRC values by 11.7%, 6.6%, and 7.9% compared to MR for the three GO aspects. Moreover, TNP and ED separately show the best and worst performances among six methods, although they use the similar metric functions (TNP uses the square of ED). These data suggest that the GO recognition accuracy can be improved via feature space mapping when coupled with triplet network learning. In addition, our result shows that MR obtains higher values of WAFmax and WAAUPRC than PCC for each GO aspect; this is consistent with the fact that MR has replaced PCC as the new co-expression measure in current co-expression databases [24].

In Figure 2C and D, we further compare the performances of the six expression profile-based methods on a subset of 98 non-coding genes in the test datasets of the 8 species, where TNP outperforms again all other five methods. Specifically, based on Fmax, TNP achieves 8.6%, 4.2%, and 8.4% improvements compared to the second-best performer for MF, BP, and CC, respectively. At the same time, the corresponding increases of AUPRC values are 7.7%, 6.5%, and 4.4%, respectively, for the three GO aspects.

**Supplementary figure S4:**
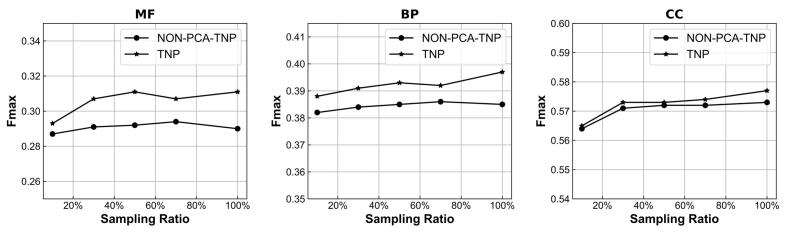
**Variation curves of Fmax values of TNP and NON-PCA-TNP on the test dataset of human species versus the sampling ratios in expression data**. NON-PCA-TNP, triplet network-based pipeline without principal component analysis.

**Supplementary figure S5:**
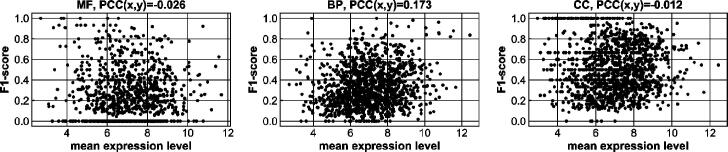
**The scattering plots of mean expression level versus F1-scores for 1470 human test genes by TNP**.

In addition, we used the human data to examine the influence of the different characteristics of gene expression data on the prediction performance of TNP. First, as illustrated in Table S6 and Figure S4 and discussed in File S5, the number of expression samples (*i.e.*, the dimension of expression profile vector) is not critical to the TNP performance. In fact, the Fmax values of TNP trained on 30% of the expression samples are only slightly (*i.e.*, by 1.3%, 1.5%, and 0.7%) lower than those trained on all the data for MF, BP, and CC, respectively. This is probably due to the inherent redundancy among different samples for the same species. To illustrate this point, we further compared TNP with and without its PCA [32] procedure, which was used to reduce redundant information of expression samples. The result showed that skipping PCA leads to a clear and consistent drop of the performance (Figure S4), which confirms the negative impact of data redundancy on the performance. Finally, we found that there is no strong correlation between the function prediction accuracy of each gene (in terms of gene-level F1-score) and its mean expression level, with a neglectable PCC value ranging from −0.026 to 0.173 for all GO aspects (Figure S5).

### Expression similarity has a closer correlation with functional similarity in the embedding feature space than in the original feature space

One important component of TNP is feature space mapping, in which the expression score calculations are transferred from the original feature space to the embedding feature space (Figure 1B and C). To examine the impact of the feature space mapping on GO prediction, we deigned and executed the following test.

For a query gene, we first rank all genes in a training dataset in descending order of the expression similarity between training gene and query, and select the top K(K=100) genes as templates. In the original feature space, the expression similarity is measured by MR, PCC, MLC, SRC, and ED, respectively. In the embedding feature space, the expression similarity for TNP is calculated by the square of the ED. Then, the weighted functional similarity (WFS), between templates and query, can be calculated as:

(12) $$WFS=\frac{\sum_{i=1}^{K}w_{i}∙FS_{i}}{\sum_{i=1}^{K}w_{i}},\;\;w_{i}=1-\left(r_{i}-1\right)/K$$

where $w_{i}$ and $r_{i}$ are the weight and rank, respectively, for i-th template, and ${\mathrm{FS}}_{i}$ is the functional similarity between the i-th template and query measured by F1-score between their experimental GO terms (see File S1A). Finally, the average weighted functional similarity (AVG_WFS) for all test genes is calculated by:

(13) $$AVG\_WFS=\sum_{i=1}^{N_{M}}{WFS}_{i}/N_{M}$$

where $N_{M}$ is the total number of test genes in the M species used here. A higher value of AVG_WFS indicates a closer correlation between expression similarity and functional similarity.

**Figure 3:**
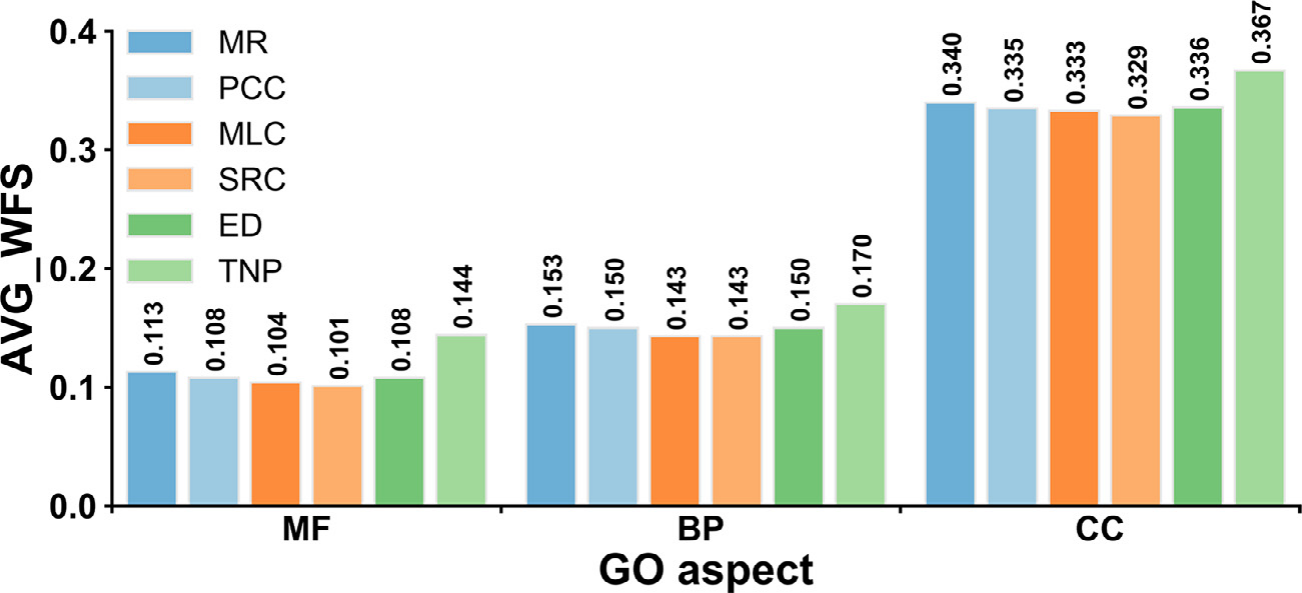
**Comparison of the AVG_WFS valuesofsix transcript EPGP methods on 8 test species** AVG_WFS, average weighted functional similarity.

**Supplementary figure S6:**
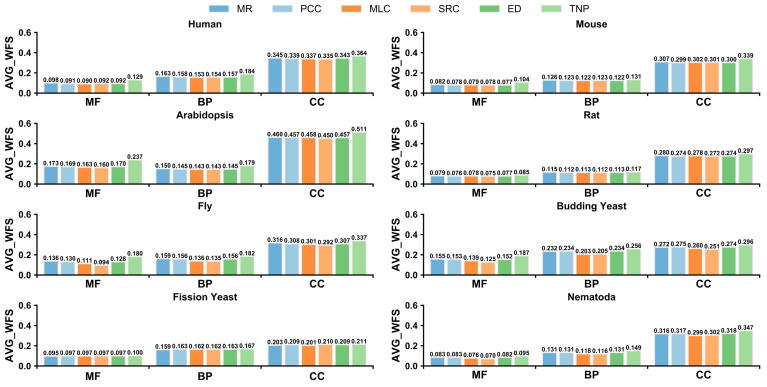
**The AVG_WFS values of six measures for three GO aspects in 8 individual species**.

Figure 3 shows the AVG_WFS values of six measures for three GO aspects in the 8 species. For each GO aspect, we found that TNP achieves the highest AVG_WFS among six measures. More specifically, the AVG_WFS values of TNP are 27.4%, 11.1%, and 7.9% higher than those of the second-best performer, MR, for MF, BP, and CC, respectively. Moreover, the AVG_WFS values of six measures in each individual species are listed in Figure S6, where TNP outperforms again other measures in all GO aspects.

**Supplementary figure S7:**
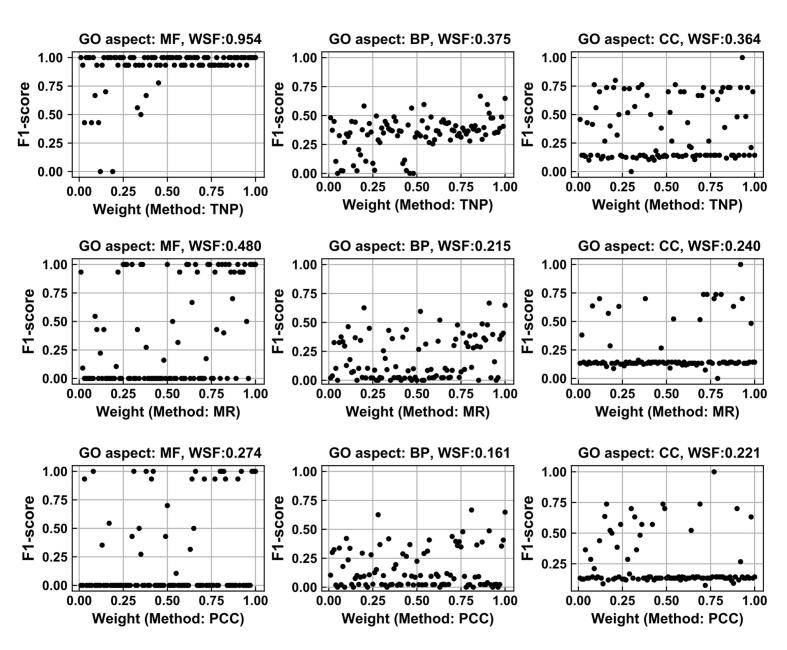
**The scattering plots of weights versus F1-scores of 100 templates for the gene *MIRLET7C* over TNP, MR, and PCC**.

As an illustrative, we listed in Figure S7 a scattering plot of F1-score *versus* weight for a non-coding gene *MIRLET7C* (Entrez ID: 406885) in the test dataset of human species. Here, we used three measures, *i.e.*, TNP, MR, and PCC, to select 100 templates with the highest expression similarity to the query. The expression similarity for different measures can be normalized as the weight [$w_{i}$ in Equation (12)]. The functional similarity is assessed by the F1-score of experimental GO terms between two genes. It can be seen that TNP achieves a higher WFS value than both MR and PCC for each GO aspect, because it selects more templates which have a higher expression similarity (or weight) and functional similarity (or F1-score) with the query than the two control measures. Since the data from TNP are directly taken from the embedding feature space after triplet network training, these results suggest that the expression similarity for TNP in the embedding feature space has a closer correlation with functional similarity compared to the other measures in the original feature space.

### Protein homology inference and triplet network-based expression make most important contributions on TripletGO prediction

**Figure 4:**
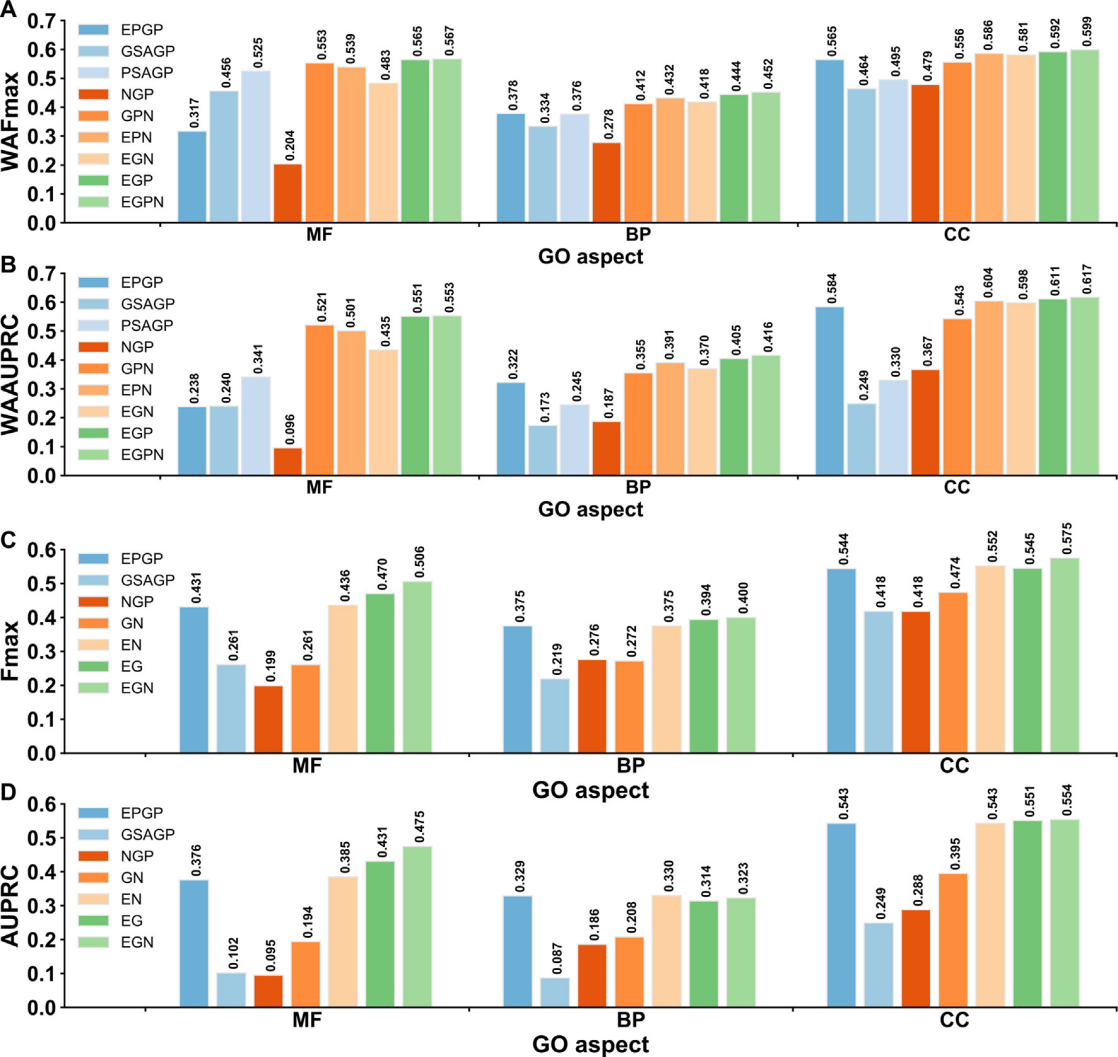
**Comparison of GO prediction results using different methods** **A.** The WAFmax values on the test datasets of 8 species for 9 GO prediction methods. **B.** The WAAUPRC values on the test datasets of 8 species for 9 GO prediction methods. **C.** The Fmax values on 98 non-coding genes for 7 GO prediction methods. **D.** The AUPRC values on 98 non-coding genes for 7 GO prediction methods. EGPN, EPGP + GSAGP + PSAGP + NGP; GPN, GSAGP + PSAGP + NGP; EPN, EPGP + PSAGP + NGP; EGN, EPGP + GSAGP + NGP; EGP, EPGP + GSAGP + PSAGP; GN, GSAGP + NPG; EN, EPGP + NPG; EG, EPGP + GSAGP.

**Supplementary figure S8:**
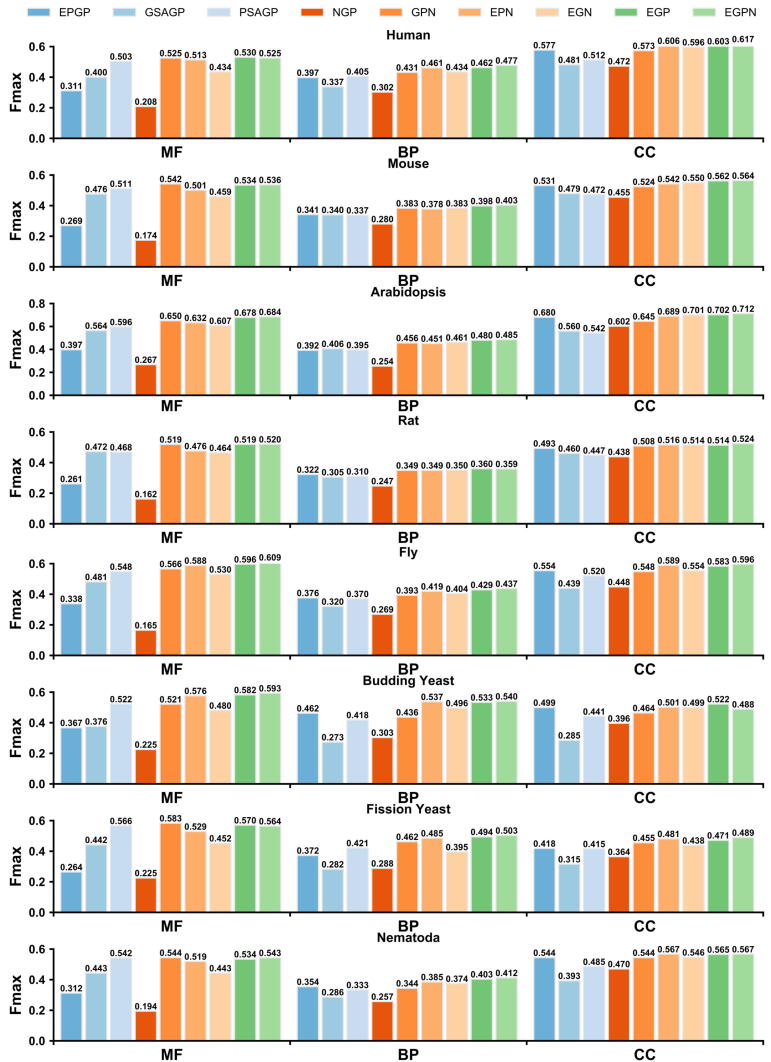
**The Fmax values of nine GO prediction methods on the test datasets for 8 individual species**.

**Supplementary figure S9:**
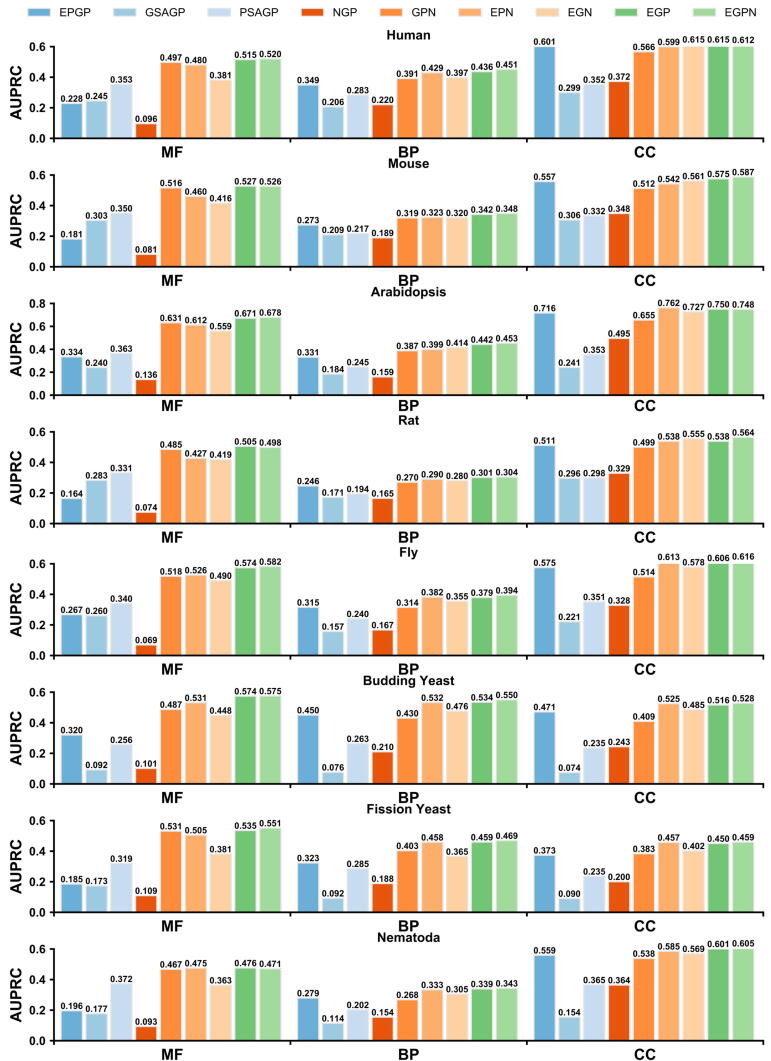
**The AUPRC values of nine GO prediction methods on the test datasets for 8 individual species**.

**Supplementary figure S10:**
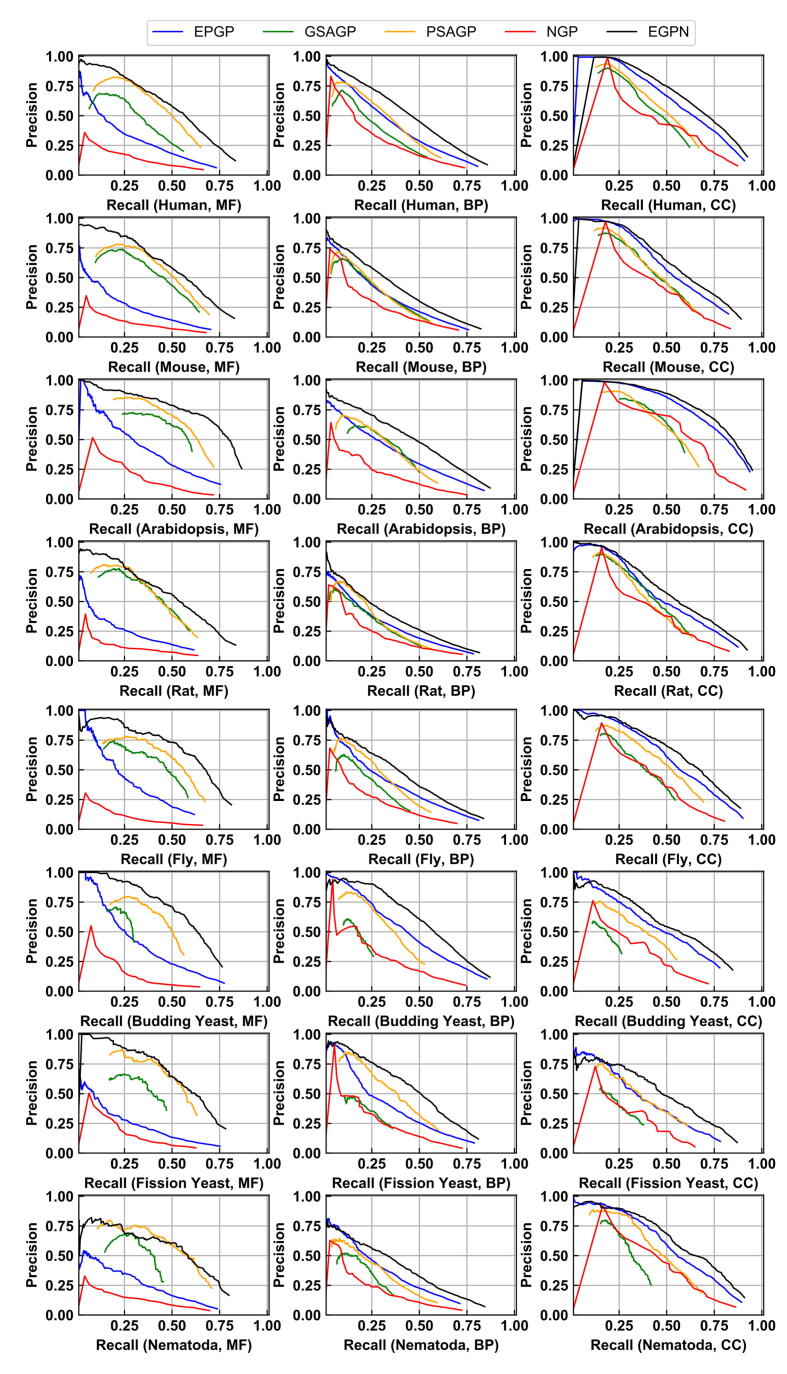
**The PRCs of five GO prediction methods on the test datasets for 8 individual species**.

To examine the contributions of four component methods in TripletGO, we compared the performances of four individual methods, including EPGP by TNP, GSAGP, PSAGP, and NGP, and five combination methods, including GSAGP + PSAGP + NGP (GPN), EPGP + PSAGP + NGP (EPN), EPGP + GSAGP + NGP (EGN), EPGP + GSAGP + PSAGP (EGP), and EPGP + GSAGP + PSAGP + NGP (EGPN = TripletGO). To be fair, we optimized the confidence scores of the combination methods using the same network in Figure 1A. Figure 4A and B list the WAFmax and WAAUPRC values of all nine methods on the test datasets of eight species, where the *P* values between EGPN and the other eight methods in Student’s *t*-test for WAFmax and WAAUPRC are listed in Table S7. In addition, the performances of all nine methods for each individual species are summarized in Figures S8–S10 and Table S8, and discussed in File S6.

From the data in Figure 4A and B and Table S7, we can conclude that each individual method contributes to improving the TripletGO prediction performance. Specifically, the WAFmax and WAAUPRC values of EGPN are much higher than the corresponding values by each of four individual methods. Importantly, the performance of EGPN is also significantly better than that of the other four combination methods. In terms of WAFmax, for example, EGPN gains 6.7%, 4.0%, 9.5%, and 1.1% average improvements for three GO aspects in comparison with GPN, EPN, EGN, and EPG, respectively. At the same time, the corresponding average increases of WAAUPRC are 12.3%, 6.3%, 14.2%, and 1.4%. The first and second largest increases are caused by adding PSAGP to EGN and adding EPGP to GPN, respectively, in BP and CC aspects. In addition, among the four individual methods, PSAGP and EPGP achieve the best performance for MF and BP/CC, respectively. These data demonstrate the importance of the protein-level homology inference and triplet network-based expression, separately, to the TripletGO prediction.

We further investigated the contributions of proposed methods for non-coding genes. Since non-coding genes have no available prediction results in PSAGP, we compared the performances of three individual gene-level methods and four combination methods, including GSAGP + NPG (GN), EPGP + NPG (EN), EPGP + GSAGP (EG), and EPGP + GSAGP + NGP (EGN = TripletGO). Figure 4C and D illustrate the Fmax and AUPRC values of seven GO prediction methods for 98 non-coding genes. The *P* values between EGN and other six methods in Student’s *t*-test for Fmax and AUPRC are shown in Table S9. Again, we can see that each of three gene-level methods helps to improve accuracy of GO prediction for non-coding genes. On the basis of Fmax, for example, EGN achieves the best performance among seven methods. Specifically, EGN gains 7.7%, 1.5%, and 4.2% improvements for MF, BP, and CC, respectively, compared to the second-best performer. With respect to AUPRC, although EGN shows a slightly lower value than EPGP and EN in BP, it achieves the best performance for MF and CC. In addition, EPGP significantly outperforms other two individual methods through all score metrices, which highlights again the importance of the transcript expression component to the TripletGO prediction.

### Comparison of TripletGO with existing gene function prediction methods

We compared TripletGO with two most recently developed gene function prediction approaches, *i.e.*, GENETICA [7] and GeneNetwork [44], which were both based on expression profiles. Different from our work, these two approaches are designed at the term-centric level. Specifically, for a GO term $Q_{i}$, each gene is labeled as “1” or “0”, where “1” means this gene is associated with $Q_{i}$ in the experimental annotation. Then, each gene is assigned with a confidence score for $Q_{i}$ using leave-one-out strategy. Finally, the area under the receiver operating characteristic curve (AUROC) is used to evaluate the prediction performance of $Q_{i}$ by combining the confidence scores and labels for all genes. In light of this, our models are compared with GENETICA and GeneNetwork by term-centric evaluation.

**Figure 5:**
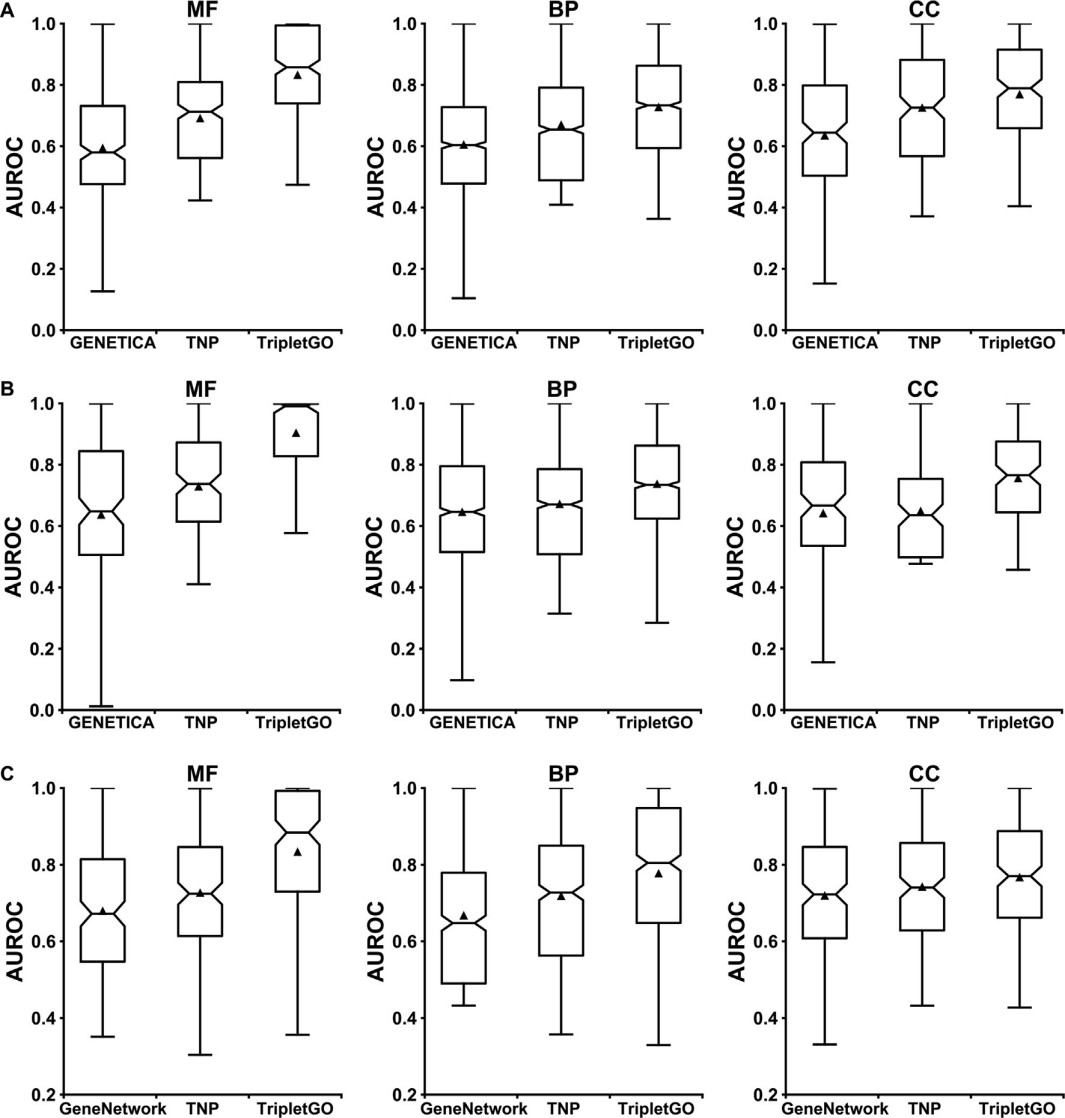
**Comparison oftheAUROC values ofdifferentgene function predictionmethods on the common dataset** **A.** GENETICA, TNP, and TripletGO on human genes. **B.** GENETICA, TNP, and TripletGO on mouse genes. **C.** GeneNetwork, TNP, and TripletGO on human genes. In each box, the median line and triangle represent the median and mean AUROC values, respectively. AUROC, area under the receiver operating characteristic curve.

**Supplementary figure S11:**
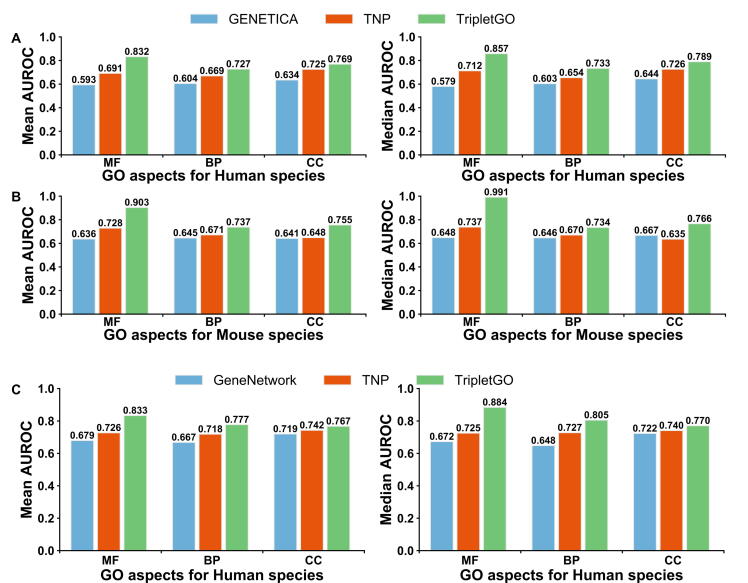
**Comparison of mean and median AUROC values of three GO aspects by different methods on the common dataset**. **A**. GENETICA, TNP, and TripletGO on human genes. **B**. GENETICA, TNP, and TripletGO on mouse genes. **C**. GeneNetwork, TNP, and TripletGO on human genes.

Between our work and GENETICA, there are 287 MF terms, 1340 BP terms, and 186 CC terms in common for human genes. As for mouse genes, there are 149, 1230, and 128 common terms for MF, BP, and CC, respectively (File S7A). Figure 5A and B plot the distributions of AUROC values by GENETICA, TNP, and TripletGO on three GO aspects in human and mouse, respectively. Figure S11A and B show the mean and median AUROC values for these three methods. While TNP and GENETICA are both expression profile-based models, the former shows a better performance than the latter. In human genes, TNP achieves 16.5%, 10.8%, 14.4% increases of the mean AUROC values for MF, BP, and CC, respectively, compared to GENETICA. At the same time, the corresponding increases of median AUROC values are 23.0%, 8.5%, and 12.7%. As for mouse, although TNP gains a slightly lower median AUROC than GENETICA in CC, it achieves significant improvements of the corresponding measures in MF and BP. In addition, TripletGO shows a significantly better performance than both TNP and GENETICA, mainly because it integrates complementary information from sources other than expression profiles.

There are 165, 522, and 182 common terms for MF, BP, and CC, respectively, in human genes between our work and GeneNetwork (File S7B). Figure 5C shows the AUROC distributions of three GO aspects for GeneNetwork, TNP, and TripletGO, respectively, and Figure S11C illustrates the mean and median AUROC values of these three models. For each GO aspect, TNP shows higher mean and median AUROC values in comparison with GeneNetwork, while TripletGO outperforms both due to the integration of additional information from sequence homology alignments and prior statistics of Gene-GOA databases.

**Supplementary figure S12:**
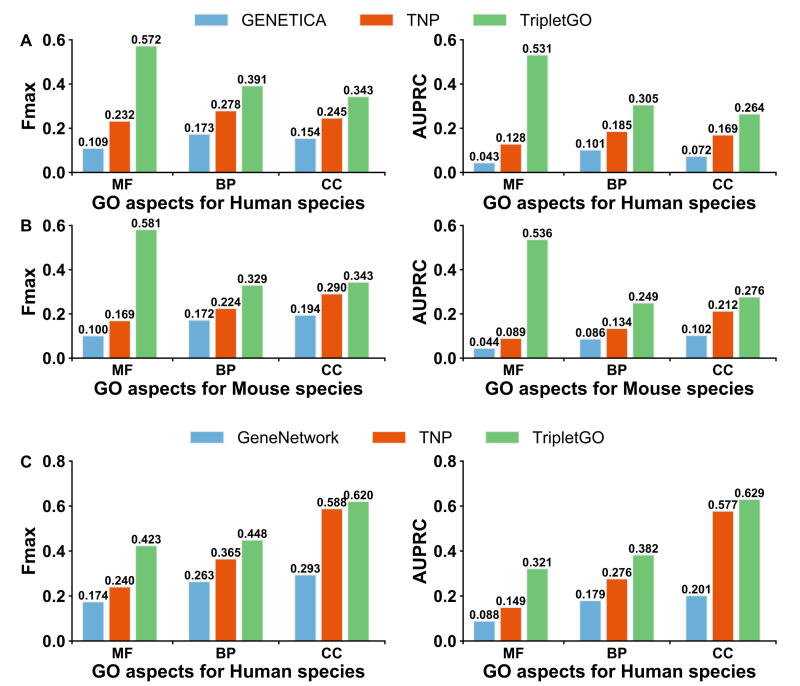
**Comparison of Fmax and AUPRC values of three GO aspects by different methods on the common dataset**. **A**. GENETICA, TNP, and TripletGO on human genes. **B**. GENETICA, TNP, and TripletGO on mouse genes. **C**. GeneNetwork, TNP, and TripletGO on human genes.

In File S7C and Figure S12, we made a further comparison of our methods with GENETICA and GeneNetwork in the gene-center level based on Fmax and AUPRC, where a similar trend (*i.e.*, TripletGO and TNP outperform the control methods) but with more significant distinctions between the methods can be seen as the term-centric comparisons.

### Testing on the CAFA3 targets

We further tested our methods on the dataset of CAFA3. The entire CAFA3 dataset consists of 66,841 training and 3328 test proteins [19] from 23 species. Since some targets have no available gene expression data, we only benchmarked our methods on the 2433 CAFA3 test proteins whose coding genes are originated from 7 species (human, mouse, *Arabidopsis*, rat, fly, budding yeast, and fission yeast) and have available expression profiles in COXPRESdb [24] or ATTED-II [21] database. It should be noted that we did not find any test proteins with expression data from nematoda species. The details of training and test datasets for the 7 species are summarized in Table S10. For each species, we randomly selected 90% training samples to re-train the TNP model and the remaining training samples were used to optimize the parameters of the model. Moreover, for GSAGP, PSAGP, and NGP, the entire CAFA3 training dataset was used to construct the corresponding template databases and prior probabilities of GO terms.

**Figure 6:**
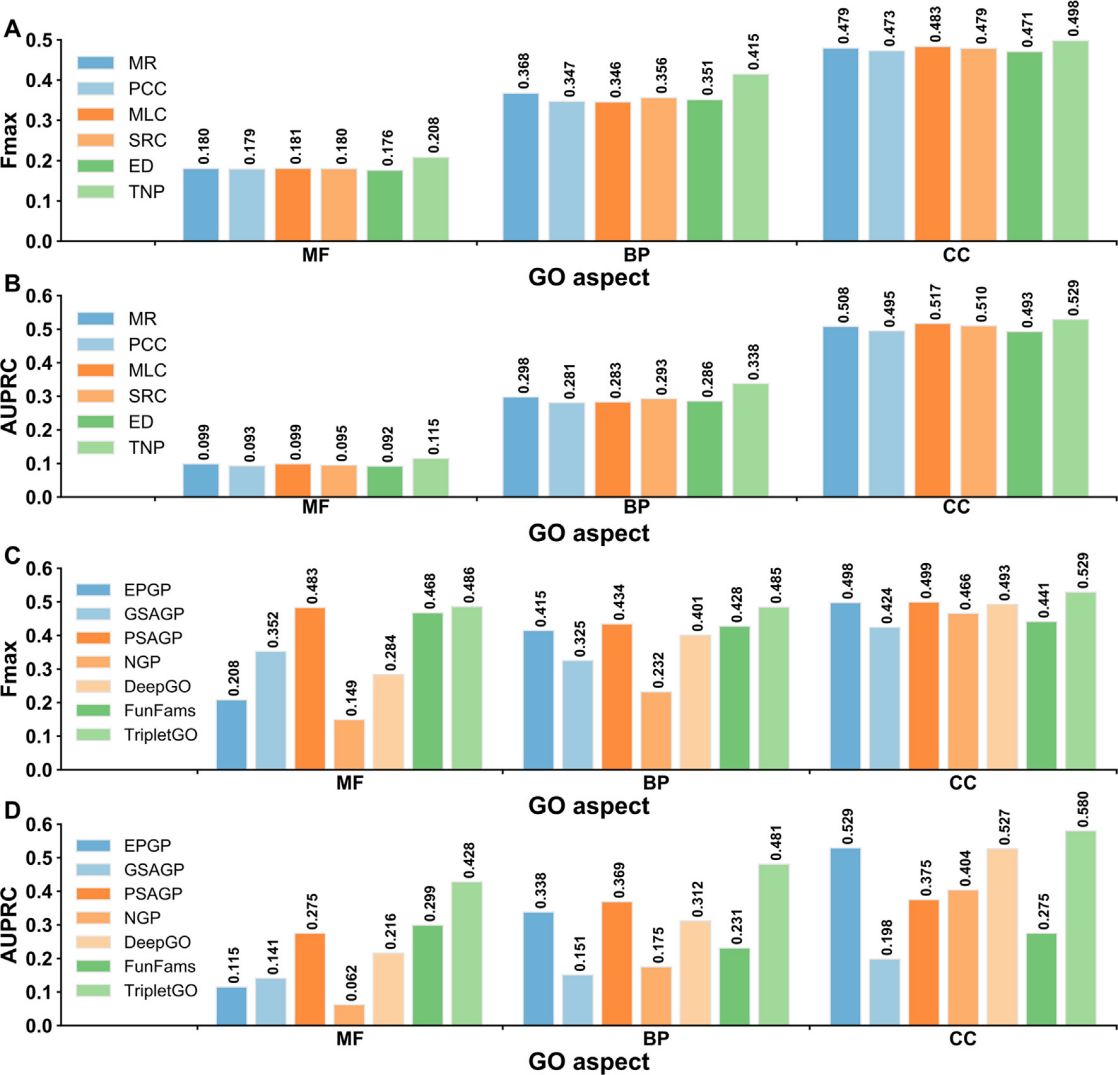
**Performance comparison on 2433 test proteins of 7 species from CAFA3 benchmark dataset** **A.** The Fmax values of 6 EPGP methods. **B.** The AUPRC values of 6 EPGP methods. **C.** The Fmax values of 5 proposed GO prediction methods and 2 existing GO prediction methods. **D.** The AUPRC values of 7 GO prediction methods.

**Supplementary figure S13:**
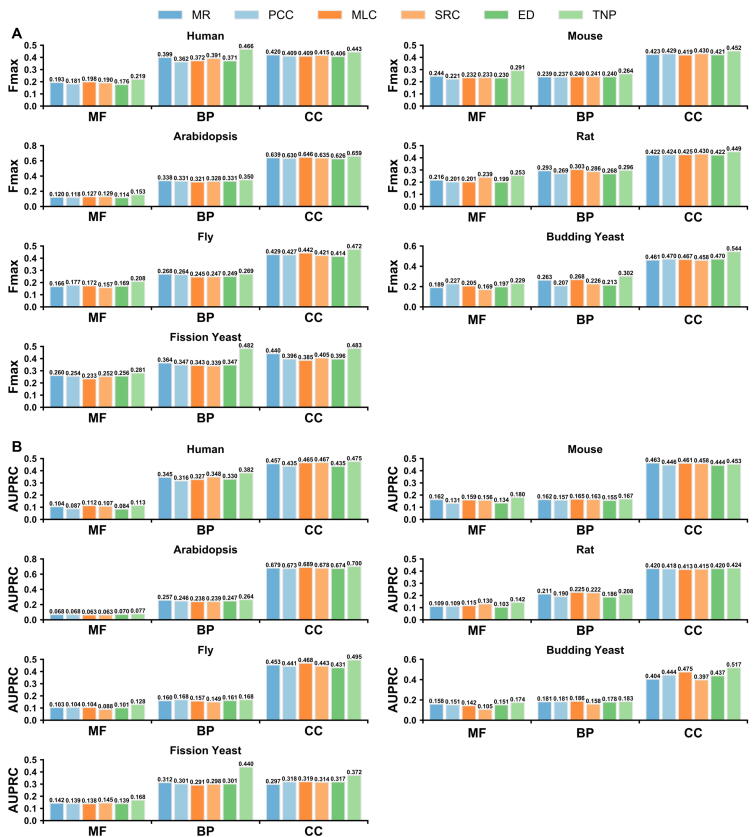
**The performance of six expression profile-based methods for 7 species on CAFA3 test dataset**. **A**. The Fmax values of six methods. **B**. The AUPRC values of six methods.

Figure 6A and B summarize the performance of six transcript EPGP methods on the 2433 test proteins, where the *P* values between TNP and the other five methods in Student’s *t*-test [43] for Fmax and AUPRC are listed in Table S11. It can be found that TNP shows better performance than other five methods for all GO aspects. Compared to the second-best performer (MR), TNP achieves 10.8% and 11.2% average improvements on three GO aspects for Fmax and AUPRC, respectively, where the *P* values are statistically significant for all the comparisons except for AUPRC values on MF (*P* = 1.41E−01) and CC (*P* = 8.01E−01). The performances of the six methods for each individual species are summarized in Figure S13 and Table S12, where TNP achieves the highest values of Fmax and AUPRC among six methods for each GO aspect in most species (see discussion in File S8).

We further benchmarked five proposed GO prediction methods, including four individual methods (*i.e.*, EPGP, GSAGP, PSAGP, and NGP) and their combination (*i.e.*, TripletGO), on the 2433 CAFA3 test proteins. In addition, we included two third-party protein function prediction methods (DeepGO [10] and FunFams [11]) which are the only methods that have downloadable programs and allow us to test independently on our selected CAFA3 dataset. Meanwhile, they represent two typical types of protocols: DeepGO is a machine learning-based method through combining convolutional neural network with protein sequence encoding, while FunFams is a template-based method and searches the functional templates using protein family information. Among them, FunFams is one of the top-performing methods and ranked at 2/4/9 positions in MF/BP/CC aspects with respect to Fmax in the CAFA3 experiment [19]. To make a fair comparison between template-based and non-template-based methods, we used the pre-set cutoff (*i.e.*, $t_{1}$ = 60% and $t_{2}$ = 30%; see Method) to exclude close homologies when running GSAGP and PSAGP; however, we did not exclude any homologies for the third-party programs and ran them under the default setting.

Figure 6C and D summarize the performance of seven GO prediction methods, where the *P* values between TripletGO and the other six methods in Student’s *t*-test [43] are listed in Table S13. We found that the composite GO prediction method, *i.e.*, TripletGO, achieves a significantly better performance than other six GO prediction methods in all GO aspects, including both the third-party (FunFams and DeepGO) methods and the component methods of TripletGO, demonstrating again the advantage of integrating gene expression and sequence profile-based approach to function prediction.

### Case studies for different GO prediction methods

**Figure 7:**
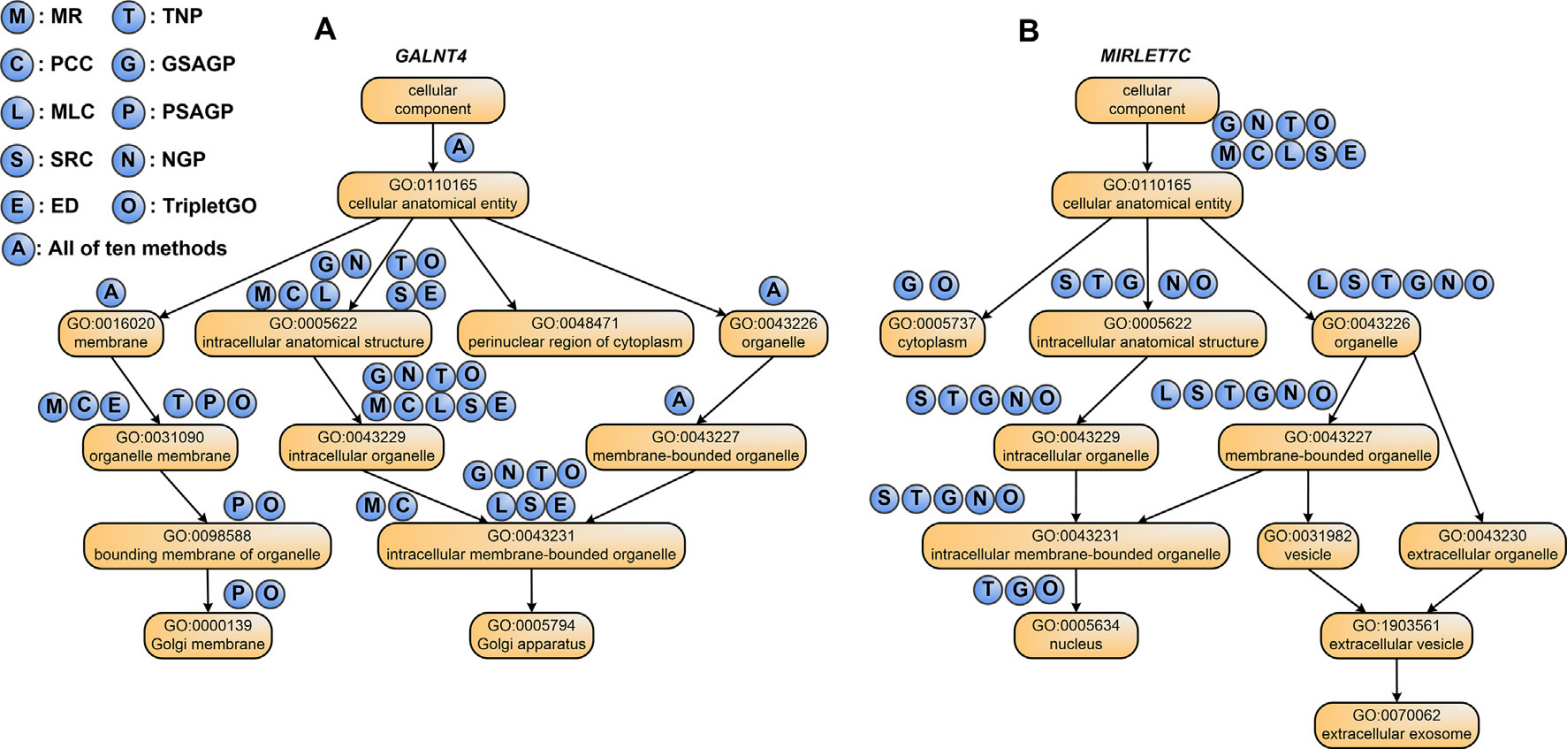
**The directed acyclic graphs of 12 GO terms in the experimental annotation on two illustrative genes** **A.** The directed acyclic graph for *GALNT4*. **B.** The directed acyclic graph for *MIRLET7C*. The circles above each GO term refer to the prediction methods, where a circle filled with “X” on a GO term “Y” indicates that method “X” can correctly predict term “Y”.

As illustrations, we selected two genes from the human genome: *GALNT4* (a protein-coding gene, Entrez ID: 8693) and *MIRLET7C* (a non-coding gene, Entrez ID: 406885), to examine the effects of different GO prediction methods. Here, each gene is associated with 12 GO terms for CC aspect from experimental annotations. Table 1 summarizes the numbers of correctly predicted GO terms (*i.e.*, true positives) and mistakenly predicted terms (*i.e.*, false positives) in CC aspect for the two genes by ten different methods, including six individual gene expression-based methods (MR, PCC, MLC, SRC, ED, and TNP), a gene sequence alignment-based method (GSAGP), a protein sequence alignment-based method (PSAGP), a naïve-based approach (NGP), and a composite approach (TripletGO). Figure 7 plots the directed acyclic graphs of GO terms in the experimental annotation and the correctly predicted GO terms of ten methods for the two genes. Moreover, the incorrectly predicted GO terms (*i.e.*, false positives) of each method are listed in Table 2. It should be noted that the predicted GO terms of each method are determined by its own cut-off value to achieve the highest Fmax value.

**Table 1:** **The modeling results of ten GO prediction methods on two illustrative genes**

**Gene**	**Measure**	**MR**	**PCC**	**MLC**	**SRC**	**ED**	**TNP**	**GSAGP**	**PSAGP**	**NGP**	**TripletGO**
*GALNT4*	NTP	8	8	7	7	8	8	7	7	7	**10**
	NFP	3	1	2	3	1	**0**	3	4	4	**0**
*MIRLET7C*	NTP	1	1	3	6	1	7	**8**	0	6	**8**
	NFP	2	2	2	2	2	1	**0**	**0**	2	**0**

*Note*: Best performers are highlighted in bold in each category. GO, Gene Ontology; MR, mutual rank; PCC, Pearson correlation coefficient; MLC, metric learning for co-expression; SRC, Spearman rank correlation; ED, Euclidean distance; TNP, triplet network pipeline; GSAGP, genetic sequence alignment-based GO prediction; PSAGP, protein sequence alignment-based GO prediction; NGP, naïve-based GO prediction; NTP, number of true positives; NFP, number of false positives.

**Table 2:** **The incorrectly predicted GO termsoften GO prediction methods on two illustrative genes**

**Method**	***GALNT4***	***MIRLET7C***
MR	GO:0005654; GO:0005829; GO:0032991	GO:0016020; GO:0005886
PCC	GO:0005829	GO:0016020; GO:0005886
MLC	GO:0005829; GO:0032991	GO:0016020; GO:0005886
SRC	GO:0005654; GO:0005829; GO:0005886	GO:0016020; GO:0005886
ED	GO:0005829	GO:0016020; GO:0005886
TNP		GO:0005654
GSAGP	GO:0005654; GO:0005829; GO:0005886	
PSAGP	GO:0031410; GO:0030133; GO:0097708; GO:0031982	
NGP	GO:0005829; GO:0032991; GO:0005634; GO:0005886	GO:0016020; GO:0032991
TripletGO		

*Note*: Incorrectly predicted GO terms are false positives.

Several interesting observations can be made from the data. First, among six gene expression-based methods, TNP can correctly recognize the most GO terms with the least false positives for each gene. Moreover, all true positives for other five methods can be effectively identified by TNP. More importantly, for *MIRLET7C*, TNP correctly recognizes 1 additional GO term, *i.e.*, GO:0005634, which is missed by the other five methods. This observation shows that TNP can predict gene function in a more precise level, because it successfully identifies some children GO terms, in which other expression-based methods fail.

Second, the combination of complementary methods increases both coverage and accuracy of the TripletGO models. For *GALNT4* (Figure 7A), the four component methods (TNP, GSAGP, PSAGP, and NGP) hit 10 true positives in total, which is more than that by each individual method, indicating that the component methods derive complementary information from different sources. By taking the combination, TripletGO achieves the highest coverage with 10 true positives and the lowest false positive rate (0 false positive). Sometimes, one component method can cover all true positives predicted by other methods. For example, for *MIRLET7C* (Figure 7B), all true positives of TNP and NGP are covered by GSAGP. Even in this case, the final TripletGO accuracy is not degraded by the inclusion of a less accurate method, where TripletGO shares the same performance with the best individual method by GSAGP.

**Supplementary figure S14:**
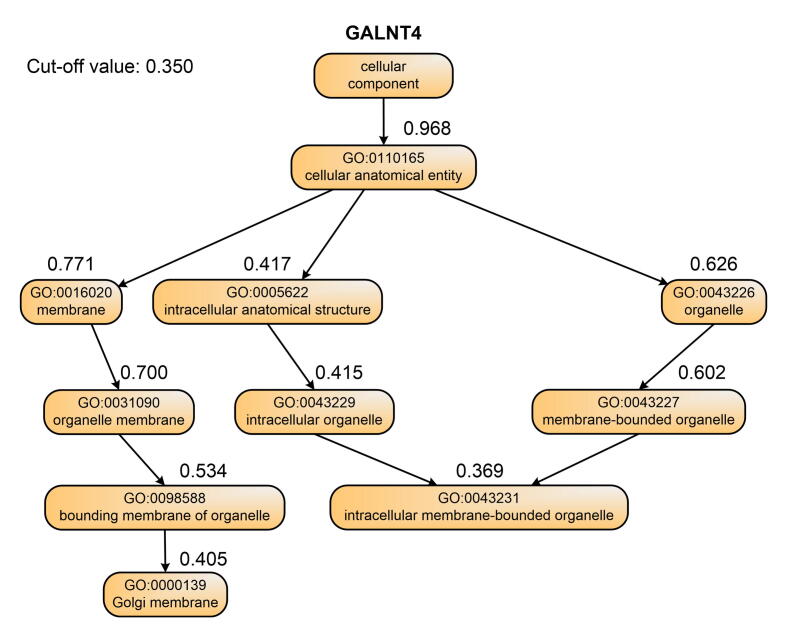
**The directed acyclic graph of predicted GO terms with corresponding confidence scores for gene GALNT4 by TripletGO**.

In addition, to further explain what is considered as a positive prediction regarding the hierarchy, we choose *GALNT4* as an illustrative example and list the confidence scores of all the candidate GO terms by TripletGO in Table S14, where the 10 GO terms whose confidence scores are higher than the cut-off value (0.35) have been predicted as positives. In Figure S14, we plot the directed acyclic graph of the 10 predicted GO terms with corresponding confidence scores. It can be found that the confidence scores of the parent terms are higher than the scores of their children, following the post-processing Equation (8).

## Conclusion

We developed a new method, TripletGO, to predict the functions of both protein-coding and non-coding genes by the integration of four gene-expression and protein homology inference pipelines. The large-scale benchmark tests on 5754 non-redundant genes from a set of 8 species demonstrated that TripletGO consistently achieved significant improvements in comparison with other state-of-the-art gene function prediction methods. Detailed analysis showed that the major advantage of TripletGO stems from two aspects. First, the new triplet network-based algorithm, when coupled with feature space mapping, efficiently recognizes functional patterns from transcript expression profiles. Second, the combination of multiple complementary pipelines, especially those with protein-level homology inference and transcript expression profile, significantly improves the coverage and accuracy of the gene function annotations.

Despite the encouraging performance, there is still considerable room for further improvements. First, the TNP needs large amounts of gene expression data with GO annotation to train the prediction model. For some species, such as dog and chicken as listed in Table S1, the number of genes with GO annotation is very limited, and for many other species of interest, no such data are available. As a result, we cannot train prediction models using the TNP from expression profiles for these species. Therefore, an extended TNP model by normalizing expression profiles across different species may help solve the issue, as well as further improve the overall accuracy of the current approach. Second, the confidence scores of the four individual methods are integrated as a consensus score by a simple one-layer neural network, where an advanced machine-learning approach may help better integrate confidence scores. Meanwhile, new GO prediction methods considering other biological aspects, such as protein–protein interactions and protein/nucleic acid structures, will help improve both the coverage and accuracy of the current gene function annotation algorithms. Studies along these lines are under progress.

## Code availability

The source code has been submitted to BioCode and is available at https://ngdc.cncb.ac.cn/biocode/tools/BT007277.

## Data availability

The online server, standalone package, and all benchmark datasets and libraries are available at https://zhanggroup.org/TripletGO/.

## CRediT author statement

**Yi-Heng Zhu:** Methodology, Validation, Writing - original draft, Data curation, Software. **Chengxin Zhang:** Software, Writing - review & editing. **Yan Liu:** Writing - review & editing. **Gilbert S. Omenn:** Writing - review & editing. **Peter L. Freddolino:** Writing - review & editing. **Dong-Jun Yu:** Writing - review & editing, Supervision. **Yang Zhang:** Conceptualization, Methodology, Writing - original draft, Supervision. All authors have read and approved the final manuscript.

## Competing interests

The authors have declared no competing interests.

## Supplementary material

Supplementary File S1 **The additional definitions in TNP**. **A**. The functional similarity for genes. **B**. Distance rank-based strategy.

Supplementary File S2 **The construction procedures of databases in sequence alignment-based GO prediction methods**. **A**. The construction procedures of Gene-GOA. **B**. The construction procedures of genetic sequence database with GO annotation. **C**. The construction procedures of protein sequence database.

Supplementary File S3 **The relationship between protein sequence identity and genetic sequence identity**.

Supplementary File S4 **Performance comparison between six EPGP methods**. **A**. The procedures of GBA strategy for EPGP. **B**. The performances of six EPGP methods for each individual species.

Supplementary File S5 **Exploring the influence of the characteristics of expression data on prediction performance for human species**.

Supplementary File S6 **The performances of nine GO prediction methods for each individual species**.

Supplementary File S7 **Performance comparison between TripletGO and existing gene function prediction models**. **A**. Finding common genes and GO terms between our datasets and GENETICA’s datasets. **B**. Finding common genes and GO terms between our datasets and GeneNetwork’s datasets. **C**. Comparison with the existing gene function prediction models in gene-center level.

Supplementary File S8 **The performances of six EPGP methods for each individual species on CAFA3 test dataset**.

## References

[1] Ashburner M., Ball C.A., Blake J.A., Botstein D., Butler H., Cherry J.M. (2000). Gene Ontology: tool for the unification of biology. Nat Genet.

[2] Murali T.M., Wu C.J., Kasif S. (2006). The art of gene function prediction. Nat Biotechnol.

[3] Zhang J., Zhang Z., Chen Z., Deng L. (2019). Integrating multiple heterogeneous networks for novel lncRNA-disease association inference. IEEE/ACM Trans Comput Biol Bioinform.

[4] Peng J., Xue H., Wei Z., Tuncali I., Hao J., Shang X. (2021). Integrating multi-network topology for gene function prediction using deep neural networks. Brief Bioinform.

[5] Lane L., Argoud-Puy G., Britan A., Cusin I., Duek P.D., Evalet O. (2012). neXtProt: a knowledge platform for human proteins. Nucleic Acids Res.

[6] Franz M., Rodriguez H., Lopes C., Zuberi K., Montojo J., Bader G.D. (2018). GeneMANIA update 2018. Nucleic Acids Res.

[7] Urzúa-Traslaviña C.G., Leeuwenburgh V.C., Bhattacharya A., Loipfinger S., van Vugt M.A.T.M., de Vries E.G.E. (2021). Improving gene function predictions using independent transcriptional components. Nat Commun.

[8] Zhang C., Freddolino P.L., Zhang Y. (2017). COFACTOR: improved protein function prediction by combining structure, sequence and protein-protein interaction information. Nucleic Acids Res.

[9] Zhang C., Zheng W., Freddolino P.L., Zhang Y. (2018). MetaGO: predicting Gene Ontology of non-homologous proteins through low-resolution protein structure prediction and protein-protein network mapping. J Mol Biol.

[10] Kulmanov M., Khan M.A., Hoehndorf R., Wren J. (2018). DeepGO: predicting protein functions from sequence and interactions using a deep ontology-aware classifier. Bioinformatics.

[11] Das S., Lee D., Sillitoe I., Dawson N.L., Lees J.G., Orengo C.A. (2015). Functional classification of CATH superfamilies: a domain-based approach for protein function annotation. Bioinformatics.

[12] Gligorijević V., Renfrew P.D., Kosciolek T., Leman J.K., Berenberg D., Vatanen T. (2021). Structure-based protein function prediction using graph convolutional networks. Nat Commun.

[13] Wan C., Jones D.T. (2020). Protein function prediction is improved by creating synthetic feature samples with generative adversarial networks. Nat Mach Intell.

[14] Smaili F.Z., Tian S., Roy A., Alazmi M., Arold S.T., Mukherjee S. (2021). QAUST: protein function prediction using structure similarity search, protein interaction and functional sequence motifs. Genomics Proteomics Bioinformatics.

[15] Esteller M. (2011). Non-coding RNAs in human disease. Nat Rev Genet.

[16] Yuan Y., Bar-Joseph Z. (2019). Deep learning for inferring gene relationships from single-cell expression data. Proc Natl Acad Sci U S A.

[17] Makrodimitris S., Reinders M.J.T., van Ham R.C.H.J. (2020). Metric learning on expression data for gene function prediction. Bioinformatics.

[18] Ray S.S., Misra S. (2019). Genetic algorithm for assigning weights to gene expressions using functional annotations. Comput Biol Med.

[19] Zhou N., Jiang Y., Bergquist T.R., Lee A.J., Kacsoh B.Z., Crocker A.W. (2019). The CAFA challenge reports improved protein function prediction and new functional annotations for hundreds of genes through experimental screens. Genome Biol.

[20] Adler J., Parmryd I. (2010). Quantifying colocalization by correlation: the Pearson correlation coefficient is superior to the Mander's overlap coefficient. Cytometry A.

[21] Obayashi T., Aoki Y., Tadaka S., Kagaya Y., Kinoshita K. (2018). ATTED-II in 2018: a plant coexpression database based on investigation of the statistical property of the mutual rank index. Plant Cell Physiol.

[22] Girolami M. (2002). Mercer kernel-based clustering in feature space. IEEE Trans Neural Netw.

[23] Schroff F., Kalenichenko D., Philbin J. (2015). Facenet: a unified embedding for face recognition and clustering. Proceeding of the 28th IEEE Conference on Computer Vision and Pattern Recognition.

[24] Obayashi T., Kagaya Y., Aoki Y., Tadaka S., Kinoshita K. (2019). COXPRESdb v7: a gene coexpression database for 11 animal species supported by 23 coexpression platforms for technical evaluation and evolutionary inference. Nucleic Acids Res.

[25] UniProt Consortium (2015). UniProt: a hub for protein information. Nucleic Acids Res.

[26] Wang L., Zhang Y., Feng J. (2005). On the Euclidean distance of images. IEEE Trans Pattern Anal Mach Intell.

[27] Zhang Z., Sabuncu M.R. (2018). Generalized cross entropy loss for training deep neural networks with noisy labels. Proceedings of the 32nd International Conference on Neural Information Processing Systems.

[28] Taha A, Chen YT, Misu T, Shrivastava A, Davis L. Boosting standard classification architectures through a ranking regularizer. arXiv 2019;1901.08616.

[29] Zhou Q., Zhong B., Lan X., Sun G., Zhang Y., Zhang B. (2020). Fine-grained spatial alignment model for person re-identification with focal triplet loss. IEEE Trans Image Process.

[30] Heller M.J. (2002). DNA microarray technology: devices, systems, and applications. Annu Rev Biomed Eng.

[31] Patro S., Sahu K.K. (2015). Normalization: a preprocessing stage. arXiv.

[32] Wold S., Esbensen K., Geladi P. (1987). Principal component analysis. Chemometr Intell Lab Syst.

[33] Han J., Moraga C., Mira J., Sandoval F. (1995). From natural to artificial neural computation.

[34] Hermans A, Beyer L, Leibe B. In defense of the triplet loss for person re-identification. arXiv 2017;1703.07737.

[35] Hoffer E., Ailon N., Feragen A., Pelillo M., Loog M. (2015). Similarity-based pattern recognition.

[36] Kingma DP, Ba J. Adam: a method for stochastic optimization. arXiv 2014;1412.6980.

[37] Sayers E.W., Barrett T., Benson D.A., Bolton E., Bryant S.H., Canese K. (2011). Database resources of the national center for biotechnology information. Nucleic Acids Res.

[38] Altschul S.F., Madden T.L., Schäffer A.A., Zhang J., Zhang Z., Miller W. (1997). Gapped BLAST and PSI-BLAST: a new generation of protein database search programs. Nucleic Acids Res.

[39] Eckle K., Schmidt-Hieber J. (2019). A comparison of deep networks with ReLU activation function and linear spline-type methods. Neural Netw.

[40] Gillis J., Pavlidis P. (2013). Characterizing the state of the art in the computational assignment of gene function: lessons from the first critical assessment of functional annotation (CAFA). BMC Bioinformatics.

[41] Boyd K, Eng KH, Page CD. Area under the precision-recall curve: point estimates and confidence intervals. In: Blockeel H, Kersting K, Nijssen S, Železný F, editors. Machine learning and knowledge discovery in databases. Berlin: Springer; 2013, p.451–66.

[42] Zar J.H. (1972). Significance testing of the Spearman rank correlation coefficient. J Am Stat Assoc.

[43] Ruxton G.D. (2006). The unequal variance *t*-test is an underused alternative to Student’s *t*-test and the Mann-Whitney *U* test. Behav Ecol.

[44] Deelen P., van Dam S., Herkert J.C., Karjalainen J.M., Brugge H., Abbott K.M. (2019). Improving the diagnostic yield of exome-sequencing by predicting gene–phenotype associations using large-scale gene expression analysis. Nat Commun.

