# Supplementary material for: TripletGO: Integrating Transcript Expression Profiles with Protein Homology Inferences for Gene Function Prediction
Source: Genomics Proteomics Bioinformatics. 2022 May 11;20(5):1013–27. doi: 10.1016/j.gpb.2022.03.001 (PMC10025770; doi:10.1016/j.gpb.2022.03.001)
Supplement: Supplementary File S1 — The additional definitions in TNP. A. The functional similarity for genes. B. Distance rank-based strategy. [file mmc1.docx]

**File S1 The additional definitions in triplet-network pipeline (TNP)**

**A. The functional similarity for genes**

The functional similarity of two genes is measured by the F1-score between their experimental Gene Ontology (GO) terms. For a gene pair $\left( i,j \right)$, the F1-score between their GO terms is defined as:

$F1-score=2(pre\times rec)/(pre+rec)$, $pre=ns/n_{1}$, $rec=ns/n_{2}$ (S1)

where $ns$ is the number of same GO terms between two genes, $n_{1}$ and $n_{2}$ are the numbers of GO terms for genes $i$ and $j$, respectively.

**B. Distance rank-based strategy**

The distance rank-based strategy (DRBS) is executed on normalized embedding matrix of training genes ($\boldsymbol{U}^{n}$) and normalized embedding vector of query gene ($\boldsymbol{u}^{q}$) to obtain a confidence score vector, denoted as $\boldsymbol{s}^{t}\mathbf{=}\left( s_{1}^{t}\mathbf{,}s_{2}^{t}\boldsymbol{, \ldots,}s_{r}^{t} \right)^{T}$, where $s_{j}^{t}$ is the confidence score that query is associated with the $j$-th GO term from the view of distance rank in the embedding space.

The details of DRBS are described as follows. First, we rank $m$ training genes based the distances between the training and query genes in embedding space in ascending order. The distance between the $i$-th training gene and query gene is calculated as follows:

$d\left( i,query \right)=\sum_{k=1}^{d_{N}} \left( u_{ik}^{n}-u_{k}^{q} \right)^{2}/4$ (S2)

Then, we select top $K$ training genes which have the shortest distance with query in embedding space as templates to calculate the confidence scores of GO terms for query as follows:

$s_{j}^{t}=\frac{\sum_{k=1}^{K} w_{k}\cdot I_{k}(j)}{\sum_{k=1}^{K} w_{k}}$ (S3)

$w_{k}=1-(r_{k}-1)/K$ (S4)

where $w_{k}$ is the weight for the $k$-th template, and $r_{k}$ is the rank of the $k$-th template; $I_{k}\left( j \right)=1$, if the $k$-th template is associated with the $j$-th GO term in the experimental function annotation; otherwise, $I_{k}\left( j \right)=0$. In this work, the value of $K$ is set to be 100.
