# Supplementary material for: TripletGO: Integrating Transcript Expression Profiles with Protein Homology Inferences for Gene Function Prediction
Source: Genomics Proteomics Bioinformatics. 2022 May 11;20(5):1013–27. doi: 10.1016/j.gpb.2022.03.001 (PMC10025770; doi:10.1016/j.gpb.2022.03.001)
Supplement: Supplementary File S2 — The construction procedures of databases in sequence alignment-based GO prediction methods. A. The construction procedures of Gene-GOA. B. The construction procedures of genetic sequence database with GO annotation. C. The construction procedures of protein sequence database. [file mmc2.docx]

**File S2 The construction procedures of databases in sequence alignment-based GO prediction methods**

**A. The construction procedures of gene-level GO annotation database (Gene-GOA)**

First, we download all genes with GO annotation from National Center for Biotechnology Information (NCBI) [1]. Following the third Critical Assessment of Protein Function Annotation (CAFA) experiments [2,3], we only select the genes annotated by at least one of the eight experimental evidence codes, including inference from experiment (EXP), inference from direct assay (IDA), inference from physical interaction (IPI), inference from mutant phenotype (IMP), inference from genetic interaction (IGI), inference from expression pattern (IEP), traceable author statement (TAS), and inference by curator (IC). Moreover, to explicitly consider the hierarchical structure of GO terms, if a child term is annotated to a gene, all its direct and indirect parents, as defined by the “is_a” relation in GO database [4] (http://geneontology.org/), are also annotated. The numbers of genes annotated with GO terms for molecular function (MF), biological process (BP), and cellular component (CC) are 40,160, 63,543, and 55,448, respectively, in Gene-GOA.

**B. The construction procedures of genetic sequence database with GO annotation (GSD-GOA)**

To construct the GSD-GOA, the RNA sequences of all genes in Gene-GOA are extracted from NCBI [1]. If there is no available RNA sequence for a gene, its genomic DNA sequence is selected. In addition, we discard a few genes which have no available RNA or genomic DNA sequences in NCBI. After this, GSD-GOA includes 39,179 sequences with MF terms, 61,699 sequences with BP terms, and 5,4117 sequences with CC terms.

**C. The construction procedures of protein sequence database (PSD)**

The PSD is constructed as follows. For each gene in Gene-GOA, we map it as the corresponding coding protein sequences in UniProt database [5] using a gene–protein mapping table. After this, 78,170 genes can be mapped as 119,876 protein sequences.

**Reference**

[1] Sayers EW, Barrett T, Benson DA, Bolton E, Bryant SH, Canese K, et al. Database resources of the National Center for Biotechnology Information. Nucleic Acids Res 2011;39:D38–51.

[2] Radivojac P, Clark WT, Oron TR, Schnoes AM, Wittkop T, Sokolov A, et al. A large-scale evaluation of computational protein function prediction. Nat Methods 2013;10:221–7.

[3] Jiang Y, Oron TR, Clark WT, Bankapur AR, D’Andrea D, Lepore R, et al. An expanded evaluation of protein function prediction methods shows an improvement in accuracy. Genome Biol 2016;17:184.

[4] Harris MA, Clark J, Ireland A, Lomax J, Ashburner M, Foulger R, et al. The Gene Ontology (GO) database and informatics resource. Nucleic Acids Res 2004;32:D258-61.

[5] Uniprot Consortium. UniProt: a hub for protein information. Nucleic Acids Res 2015;43:D204–12.
