# Supplementary material for: TripletGO: Integrating Transcript Expression Profiles with Protein Homology Inferences for Gene Function Prediction
Source: Genomics Proteomics Bioinformatics. 2022 May 11;20(5):1013–27. doi: 10.1016/j.gpb.2022.03.001 (PMC10025770; doi:10.1016/j.gpb.2022.03.001)
Supplement: Supplementary File S3 — The relationship between protein sequence identity and genetic sequence identity. [file mmc3.docx]

**File S3 The relationship between protein sequence identity and genetic sequence identity**

In this work, we use three machine learning models, including liner regression (LR) [1], support vector regression (SVR) [2], and neural network with one hidden layer (NN), to fit the relationship between protein sequence identity and genetic sequence identity, as follows:

$y={f(x)}_{\mathrm{Model}}$ (S1)

where $x$ is the protein sequence identity, $y$ is the genetic sequence identity, $Model\in\{LR, SVR, NN\}$.

First, we randomly select 100,000,000 gene–gene sequence pairs from NCBI, and map each gene–gene sequence pair as a protein–protein sequence pair in UniProt database by gene–protein mapping table. Then, to reduce computation time, we remove the protein sequences and gene sequences whose lengths are more than 10,000. After this, the number of remaining gene–gene pairs (or the corresponding protein–protein pairs) is 974,447. Next, we use standard Needleman–Wunsch algorithm [3] to calculate the sequence identity for each gene–gene pair and the corresponding protein–protein pair. Each gene–gene pair and the corresponding protein–protein pair are combined as a machine learning sample. More specifically, the sequence identity of protein–protein pair is used as the feature of sample, *i.e.,* the input of machine learning model, and the sequence identity of gene–gene pair is used as the regression value of sample, *i.e.,* the output of machine learning model. Finally, we separately train LR, SVR, and NN on the 974,447 samples, and found that ${f(30\%)}_{LR}=58.6\%, {f(30\%)}_{SVM}=59.3\%, and {f(30\%)}_{NN}=60.1\%$. In addition, we randomly select 10,000 gene–gene pairs and the mapped protein–protein pairs, and then plot the corresponding sequence identities, as shown in Figure S1. For the protein–protein pairs whose sequence identities are equal to 30%, the sequence identities for the corresponding gene–gene pairs are near to 60%. In light of the above, we use 60% sequence identity as the cut-off to remove the homologous gene templates.

**Reference**

[1] Montgomery DC, Peck EA, Vining GG. Introduction to linear regression analysis. 6th ed. Hoboken: John Wiley & Sons Inc; 2021.

[2] Awad M, Khanna R. Support vector regression. In: Awad M, Khanna R, editors. Efficient Learning Machines. Berlin: Springer; 2015, p.67–80.

[3] Needleman SB, Wunsch CD. A general method applicable to the search for similarities in the amino acid sequence of two proteins. J Mol Biol 1970;48:443–53.
