# Supplementary material for: TripletGO: Integrating Transcript Expression Profiles with Protein Homology Inferences for Gene Function Prediction
Source: Genomics Proteomics Bioinformatics. 2022 May 11;20(5):1013–27. doi: 10.1016/j.gpb.2022.03.001 (PMC10025770; doi:10.1016/j.gpb.2022.03.001)
Supplement: Supplementary File S4 — Performance comparison between six EPGP methods. A. The procedures of GBA strategy for EPGP. B. The performances of six EPGP methods for each individual species. [file mmc4.docx]

**File S4 Performance comparison between six expression profile-based GO prediction methods**

**A. The procedures of guilty-by-association (GBA) strategy for expression profile-based GO prediction**

In the GBA strategy, we select the template genes which have the highest similarity with query gene in terms of expression profiles, and then use the GO terms of templates to annotate the query, as follows.

**Training stage**

In a training dataset, the expression profiles of all genes can be represented as a matrix $\boldsymbol{E}={(e_{ij})}_{m\times l}$, where the $i$-th row of $\boldsymbol{E}$ is the expression profile for the $i$-th gene and denoted as $\boldsymbol{e}_{i}={(e_{i1}, e_{i2}, \ldots, e_{il})}^{T}$, $m$ is the total number of training genes, $l$ is the number of experimental samples in microarray technology [1], and $e_{ij}$ is the expression value of the $i$-th gene on the $j$-th sample. We orderly execute z-score normalization [2] and principal component analysis (PCA) [3] on expression profile matrix $\boldsymbol{E}$ to obtain a normalized matrix $\boldsymbol{E}^{n}={(e_{ij}^{n})}_{m\times h}$, where the$i$-th row of $\boldsymbol{E}^{n}$, denoted as $\boldsymbol{e}_{i}^{n}={(e_{i1}^{n}, e_{i2}^{n}, \ldots, e_{ih}^{n})}^{T}$, is the normalized expression profile vector for the $i$-th training gene.

**Prediction stage**

For a query gene, its expression profile can be represented as a vector $\boldsymbol{e}^{q}\mathbf{=}{\mathbf{(}e_{1}^{q}\mathbf{,}e_{2}^{q}\boldsymbol{, \ldots,}e_{l}^{q}\mathbf{)}}^{T}$. First, the z-score normalization and PCA are orderly executed on the expression profile vector $\boldsymbol{e}^{q}$ to obtain a normalized vector $\boldsymbol{e}_{q}^{n}\mathbf{=}\left( e_{1}^{nq}\mathbf{,}e_{2}^{nq}\boldsymbol{, \ldots,}e_{h}^{nq} \right)^{T}$. Then, for each training gene $i$, we calculate its similarity score with query based on the normalized vector $\boldsymbol{e}_{q}^{n}$ and $\boldsymbol{e}_{i}^{n}$. Next, we rank $m$ training genes based on the similarity scores in descending order. Finally, we select the top $K$ training genes as templates to annotate the GO terms of query. Specifically, the confidence score that the query is associated with GO term $Q_{j}$ can be calculated as follows:

${S\left( Q_{j} \right)}_{GBA}=\frac{\sum_{k=1}^{K} w_{k}\cdot I_{k}(Q_{j})}{\sum_{k=1}^{K} w_{k}}$ (S1)

$w_{k}=1-(r_{k}-1)/K$ (S2)

where $w_{k}$ is the weight for the $k$-th template, and $r_{k}$ is the rank of the $k$-th template; $I_{k}\left( Q_{j} \right)=1$, if the $k$-th template is associated with $Q_{j}$ in the experimental annotation; otherwise, $I_{k}\left( Q_{j} \right)=0$.

In this work, the similarity score of expression profiles between two genes are measured by four unsupervised methods, including Pearson correlation coefficient (PCC) [4], Spearman rank correlation (SRC) [5], mutual rank (MR) [6], and Euclidean distance (ED) [7], and a recently proposed supervised method, *i.e.*, metric learning for co-expression (MLC) [8].

The PCC between the $i$-th training gene and query gene is calculated as follows:

$\mathrm{PCC}\boldsymbol{(e}_{i}^{n}\boldsymbol{,e}_{q}^{n}\boldsymbol{)=}\frac{\sum_{j=1}^{h} \boldsymbol{(}e_{ij}^{n}-\overline{\boldsymbol{e}_{i}^{n}}\boldsymbol{)\cdot(}e_{j}^{nq}-\overline{\boldsymbol{e}_{q}^{n}}\boldsymbol{)}}{\sqrt{\sum_{i=1}^{h} {\boldsymbol{(}e_{ij}^{n}-\overline{\boldsymbol{e}_{i}^{n}}\boldsymbol{)}}^{2}}\boldsymbol{\cdot}\sqrt{\sum_{i=1}^{h} {\boldsymbol{(}e_{j}^{nq}-\overline{\boldsymbol{e}_{q}^{n}}\boldsymbol{)}}^{2}}}$ (S3)

where $\overline{\boldsymbol{e}_{i}^{n}}$ and $\overline{\boldsymbol{e}_{q}^{n}}$ are mean values for $\boldsymbol{e}_{i}^{n}$ and $\boldsymbol{e}_{q}^{n}$, respectively.

The SRC between the $i$-th training gene and query gene is calculated as follows:

$\mathrm{SRC}\boldsymbol{(e}_{i}^{n}\boldsymbol{,e}_{q}^{n}\boldsymbol{)=}1-\frac{6\sum_{j=1}^{h} {(r_{ij}-r_{j})}^{2}}{h(h^{2}-1)}$ (S4)

where $r_{ij}$ is rank of $e_{ij}^{n}$ in the elements of $\boldsymbol{e}_{i}^{n}$ in ascending order, and $r_{j}$ is the rank of $e_{j}^{nq}$ in the elements of $\boldsymbol{e}_{q}^{n}$ in ascending order.

Due to the long computation time of MR values, we directly download MR values of genes from COXPRESdb [9] and ATTED-II databases [6]. In a species with $M$ genes, the MR value between gene $i$ and gene $j$ is calculated as follows. First, we calculate the PCC values between gene $i$ and the remaining $M-1$ genes based on the corresponding expression profile vectors, and rank the $M-1$ genes based on the PCC values in descending order. Similarly, we calculate the PCC values between gene $j$ and the remaining $M-1$ genes, and rank the $M-1$ genes in descending order based on PCC values. Then, the MR value between genes $i$ and $j$ can be calculated:

$MR\left( i,j \right)=\sqrt{rank(i)\cdot rank(j)}$ (S5)

where $rank(i)$ is the rank of gene $i$ in $M-1$ genes for gene $j$, and $rank(j)$ is the rank of gene $j$ in $M-1$ genes for gene $i$.

The ED between the $i$-th training gene and query gene is calculated as follows:

$\mathrm{ED}(\boldsymbol{e}_{i}^{n}\boldsymbol{,e}_{q}^{n})=\sqrt{\sum_{j=1}^{h} \left( e_{ij}^{n}-e_{j}^{nq} \right)^{2}}$ (S6)

In MLC, the similarity between the $i$-th training gene and query gene is measured by weight inner product (WIP) as follows:

$\mathrm{WIP}(\boldsymbol{e}_{i}^{n}\boldsymbol{,e}_{q}^{n})={\boldsymbol{(e}_{i}^{n})}^{T}\cdot W\cdot\boldsymbol{e}_{q}^{n}$ (S7)

where $W=diag\left( w \right)$ is a diagonal matrix and can be optimized by the Broyden-Fletcher-Goldfarb-Shanno method [10].

The higher values of PCC, SRC, and WIP indicate the higher similarity, while the lower values of MR and ED mean the higher similarity.

**B. The performances of six expression profile-based GO prediction methods for each individual species**

For each of 8 species, we will evaluate the performances of six expression profile-based GO prediction methods on the corresponding test dataset. For each method, we execute it 10 times and then use the average of all prediction results as the final result.

Figure S2 show the values of maximum F1-score (Fmax) and area under the precision-recall curve (AUPRC) for 8 species via six expression profile-based methods. Table S5 summarizes the *P* values of Fmax and AUPRC values between TNP and other five methods in Student’s *t*-test [11] for 8 species. In comparison between TNP and MLC, we use two samples *t*-test [12] to calculate *P* value due to that the prediction results in 10 times are different for MLC/TNP. In comparison between TNP and PCC, MR, SRC, ED, we use single samples *t*-test [13] to calculate *P* value, because the prediction results in 10 times are same for PCC/MR/SRC/ED. From Figure S2 and Table S5, we can observe that TNP achieves the highest values of Fmax and AUPRC among six methods for each GO aspect in each species. For example, in human species, the improvements of Fmax values between TNP and MR are 12.7%, 8.2%, 3.8%, respectively, with *P* values of 1.29×10^-04^, 7.83×10^-09^, and 5.07×10^-07^ for MF, BP, and CC aspects. As another example, the average improvement of AUPRC values of three GO aspects between TNP and the second best performer is 8.6% with *P* values < 0.05 for Arabidopsis species.

Figure S3 plots the precision-recall curves (PRC) of six expression profile-based methods for three GO aspects in 8 species. For each GO aspect in each species, we can find that TNP has the highest precision values among six expression profile-based methods at all different recall rates.

**Reference**

[1] Heller MJ. DNA microarray technology: devices, systems, and applications. Annu Rev Biomed Eng 2002;4:129–53.

[2] Patro SGK, Sahu KK. Normalization: a preprocessing stage. arXiv 2015; https://doi.org/10.48550/arXiv.1503.06462.

[3] Wold S, Esbensen K, Geladi P. Principal component analysis. Chemometr Intell Lab Syst 1987;2:37–52.

[4] Adler J, Parmryd I. Quantifying colocalization by correlation: the Pearson correlation coefficient is superior to the Mander’s overlap coefficient. Cytometry A 2010;77:733–42.

[5] Zar JH. Significance testing of the Spearman rank correlation coefficient. J Am Stat Assoc 1972;67:578–80.

[6] Obayashi T, Aoki Y, Tadaka S, Kagaya Y, Kinoshita K. ATTED-II in 2018: a plant coexpression database based on investigation of the statistical property of the mutual rank index. Plant Cell Physiol 2018;59:e3.

[7] Wang L, Zhang Y, Feng J. On the Euclidean distance of images. IEEE Trans Pattern Anal Mach Intell 2005;27:1334–9.

[8] Makrodimitris S, Reinders MJT, van Ham RCH. Metric learning on expression data for gene function prediction. Bioinformatics 2020;36:1182-90.

[9] Obayashi T, Kagaya Y, Aoki Y, Tadaka S, Kinoshita K. COXPRESdb v7: a gene coexpression database for 11 animal species supported by 23 coexpression platforms for technical evaluation and evolutionary inference. Nucleic Acids Res 2019;47:D55–62.

[10] Byrd RH, Lu P, Nocedal J, Zhu C. A limited memory algorithm for bound constrained optimization. SIAM J Sci Comput 1995;16:1190-208.

[11] Ruxton GD. The unequal variance *t*-test is an underused alternative to Student’s *t*-test and the Mann–Whitney U test. Behav Ecol 2006;17:688–90.

[12] Heeren T, D’Agostino R. Robustness of the two independent samples t‐test when applied to ordinal scaled data. Stat Med 1987;6:79-90.

[13] Crawford J, Howell DC, Garthwaite PH. Payne and Jones revisited: estimating the abnormality of test score differences using a modified paired samples t test. J Clin Exp Neuropsychol 1998;20:898-905.
