# Supplementary material for: TripletGO: Integrating Transcript Expression Profiles with Protein Homology Inferences for Gene Function Prediction
Source: Genomics Proteomics Bioinformatics. 2022 May 11;20(5):1013–27. doi: 10.1016/j.gpb.2022.03.001 (PMC10025770; doi:10.1016/j.gpb.2022.03.001)
Supplement: Supplementary File S5 — Exploring the influence of the characteristics of expression data on prediction performance for human species. [file mmc5.docx]

**File S5 Exploring the influence of the characteristics of expression data on prediction performance for human species**

We used the human data to explore the impact of the characteristics of expression data on the prediction performance of TNP from the following two aspects.

First, to analyze the dependency of the prediction performance of TNP on the number of expression samples, we deigned the following test. In human benchmark dataset, each gene is associated with 27,655 expression samples, as shown in Table S1. For each human gene, we randomly extracted 10%, 30%, 50%, and 70% expression samples, respectively. This generates four sub-datasets from the original human benchmark set. Each sub-dataset has the same genes but different numbers (*i.e.,* 2765, 8296, 13,827, and 19,358) of expression samples. In each sub-benchmark dataset, we re-trained the TNP model on the training dataset, and then evaluated the corresponding prediction performance on the test dataset. Moreover, to eliminate the redundant information of expression samples, TNP uses the PCA to reduce the dimension of expression profile vector. To further demonstrate the effectiveness of PCA, we added a comparison model, named NON-PCA-TNP, which removes the PCA from TNP.

Table S6 summarizes the Fmax values of TNP and NON-PCA-TNP on the test dataset of human species for different sampling ratios in expression data. Figure S4 plots the variation curves of Fmax values of two methods versus the sampling ratios. From Table S6 and Figure S4, the following two conclusions can be drawn. First, the performance of TNP has a low dependency on the number of expression samples. Specifically, with the increase of sampling ratio from 10% to 100%, the performance of TNP is slowly improved. Under the sampling ratio of 30%, TNP can achieve the acceptable Fmax values, which are only decreased on average by 1.2% on three GO aspects in comparison with the model using all expression samples. Second, the PCA is helpful for relieving information redundancy among expression samples. Concretely, we found that the performance of TNP is consistently better than that of NON-PCA-TNP under all sampling ratios for each GO aspect.

In addition, to analyze the relation between prediction performance and expression level for individual gene, we further deigned the following test. For each test gene in human species, we firstly calculated the mean expression level of all expression samples and the F1-score between the predicted GO terms by TNP and actual GO terms in native annotation. Then, the PCC between mean expression level and F1-score for all test genes was calculated.

Figure S5 plots the scattering plots of mean expression level versus F1-score for 1470 human test genes by TNP, where the PCC value between mean expression level and F1-score is listed at the top. The PCC between mean expression level and F1-score ranges from $-$0.026 to 0.173. Therefore, we can conclude that there is no clear correlation between expression level and prediction performance of TNP for individual genes.
