# Supplementary material for: TripletGO: Integrating Transcript Expression Profiles with Protein Homology Inferences for Gene Function Prediction
Source: Genomics Proteomics Bioinformatics. 2022 May 11;20(5):1013–27. doi: 10.1016/j.gpb.2022.03.001 (PMC10025770; doi:10.1016/j.gpb.2022.03.001)
Supplement: Supplementary File S6 — The performances of nine GO prediction methods for each individual species. [file mmc6.docx]

**File S6 The performances of nine GO prediction methods for each individual species**

For each of 8 species, we will compare the performances of four individual methods (*i.e.,* expression profile-based GO prediction (EPGP), genetic sequence alignment-based GO prediction (GSAGP), protein sequence alignment-based GO prediction (PSAGP), and Naïve-based GO prediction (NGP)) and five combination methods (*i.e.,* GSAGP + PSAGP + NGP (GPN), EPGP + PSAGP + NGP (EPN), EPGP + GSAGP + NGP (EGN), EPGP + GSAGP + PSAGP (EGP), and EPGP + GSAGP + PSAGP + NGP (EGPN)) on the corresponding test dataset. For each combination method, we execute it 10 times and then use the average of all prediction results as the final result.

Figures S8 and S9 illustrate the values of Fmax and AUPRC for nine GO prediction methods in 8 species. Table S8 show the *P* values of Fmax and AUPRC values between EGPN and other eight methods in student’s t-test [1] for 8 species. In comparison between EGPN and four combination methods (*i.e.,* GPN, EPN, EGN, and EGP), we use two samples t-test [2] to calculate *P* value due to that the prediction results in 10 times are different for them. In comparison between EGPN and four individual methods (*i.e.,* EPGP, GSAGP, PSAGP, and NGP), we use single samples t-test [3] to calculate *P* value. From Figures S8 and S9 and Table S8, we can find the Fmax and AUPRC values of EGPN are much higher than that of four individual methods for each species. Moreover, from the view of Fmax, in species of Arabidopsis and fly, EGPN achieves the better performance than other four combination methods for each GO aspect; in species of human, mouse, rat, and nematoda, EGPN occupies one of the top two positions among five combination methods for each GO aspect; as for the remaining two species, EGPN shows the best performance in MF/BP for budding yeast and BP/CC for fission yeast. These observations further demonstrate that each individual method contributes to improving prediction performance.

Figure S10 plots the PRC of four individual methods and EPGN for three GO aspects in 8 species. For each GO aspect in each species, we can observe that the PRC of EGPN is continuously higher than that of four individual methods.

**Reference**

[1] Ruxton GD. The unequal variance t-test is an underused alternative to Student’s t-test and the Mann–Whitney U test. Behav Ecol 2006;17:688–90.

[2] Heeren T, D'Agostino R. Robustness of the two independent samples t‐test when applied to ordinal scaled data. Stat Med 1987;6:79–90.

[3] Crawford J, Howell DC, Garthwaite PH. Payne and Jones revisited: estimating the abnormality of test score differences using a modified paired samples t-test. J Clin Exp Neuropsychol 1998;20:898–905.
