# Supplementary material for: TripletGO: Integrating Transcript Expression Profiles with Protein Homology Inferences for Gene Function Prediction
Source: Genomics Proteomics Bioinformatics. 2022 May 11;20(5):1013–27. doi: 10.1016/j.gpb.2022.03.001 (PMC10025770; doi:10.1016/j.gpb.2022.03.001)
Supplement: Supplementary File S7 — Performance comparison between TripletGO and existing gene function prediction models. A. Finding common genes and GO terms between our datasets and GENETICA’s datasets. B. Finding common genes and GO terms between our datasets and GeneNetwork’s datasets. C. Comparison with the existing gene function prediction models in gene-center level. [file mmc7.docx]

**File S7 Performance comparison between TripletGO and existing gene function prediction models**

**A. Finding common genes and GO terms between our datasets and GENETICA’s datasets**

In the web page (http://genetica-network.com), GENETICA provides the prediction scores and real labels of three GO aspects for 19,635 genes in human species and 18,425 genes in mouse species. Specifically, in human species, each gene is associated with the prediction scores and real labels of 843 MF, 4203 BP, and 528 CC GO terms. As for mouse species, each gene is associated with the prediction scores and real labels of 833 MF, 4188 BP, and 525 CC terms.

In GENETICA’s datasets, 879 genes, 1176 genes, and 1241 genes for MF, BP, and CC aspects, respectively, can be found in our test dataset for human species; 572 genes, 880 genes, and 737 genes for three GO aspects can be separately found in our test dataset for mouse species. The GO terms in GENETICA and our work are represented as GO names and GO Identity documents (IDs), respectively. Due to the different versions of GO databases, only 738 MF, 3980 BP, and 476 CC GO names in GENETICA’s can be correctly mapped as the corresponding GO IDs in our dataset for human species; As for mouse species, there are 727 MF, 3965 BP, and 474 CC terms in common between our work and GENETICA. Moreover, we only consider the GO terms whose frequencies are more than 20 both in our training datasets and GENETICA’s datasets. After this, there are 879 genes annotated with 287 MF terms, 1176 genes with 1340 BP terms, and 1241 genes with 186 CC terms for human species in common between our test dataset and GENETICA’s dataset; As for mouse species, there are 572 genes with 149 MF terms, 880 genes with 1230 BP terms, and 737 genes with 128 CC terms in common. For each GO term $Q_{i}$, all genes are assigned with the prediction scores and real labels. Specifically, if a gene is associated with $Q_{i}$ both in our test dataset and GENETICA’s dataset, we label it as “1”; otherwise, it is labeled as “0”.

**B. Finding common genes and GO terms between our datasets and GeneNetwork’s datasets**

In the web page (https://www.genenetwork.nl/), we can use command “GET https://www.genenetwork.nl/api/v1/gene/geneName?db=database” to download the GO information file generated by GeneNetwork for each query gene in each GO aspect. The information file contains all GO terms in the experimental function annotation and 100 predicted GO terms with scores for a query. GeneNetwork provides all of the information files in three GO aspects for 56,435 genes.

In GeneNetwork’s dataset, 918 genes, 1230 genes, and 1328 genes for MF, BP and CC aspects, respectively, can be found in our test dataset for human species. Moreover, there are 655 MF, 2776 BP, and 536 CC terms in common between our work and GeneNetwork. In this work, we only consider the GO terms whose frequencies are more than 20 both in our training dataset and GeneNetwork’s dataset. After this, there are 918 genes associated with 165 MF terms, 1230 genes with 522 BP terms, and 1328 genes with 182 CC terms, in common, for human species between our test dataset and GeneNetwork’s dataset. For each GO term $Q_{i}$, all genes are assigned with the prediction scores and real labels. Specifically, if a gene is associated with $Q_{i}$ both in our test dataset and GeneNetwork’s dataset, we label it as “1”; otherwise, it is labeled as “0”.

**C. Comparison with the existing gene function prediction models in gene–center level.**

We further compared our methods (TNP and TripletGO) with the existing gene function predictors (GENETICA and GeneNetwork) in gene-center level. In GENETICA and GeneNetwork, the numerical distributions of prediction scores of genes are different for each GO aspect. For example, in MF aspect, the prediction scores of GO terms for all human genes by GENETICA are range from 0.618167 to 15.79360; in BP aspect, the prediction scores by GENETICA are range from 0.61976 to 19.75050. Therefore, we firstly normalize the prediction scores in the range of 0 to 1 for each GO aspect using min-max normalization. Specifically, for each GO aspect, the prediction score $S_{i}$ is normalized as:

$S_{i}^{n}=\frac{S_{i}-S_{min}}{S_{max}-S_{min}}$ (S1)

where $S_{max}$ and $S_{min}$ are the max and min values, respectively, in all prediction scores.

Based on the normalized prediction scores, the prediction performances for GENETICA and GeneNetwork can be transformed from term-center metric (the area under receiver operating characteristic curve (AUROC)) to gene-center metric (Fmax and AUPRC). Figure S12A and B shows the Fmax and AUPRC values of TNP, TripletGO, and GENETICA in human species (879 genes with 287 MF terms, 1176 genes with 1340 BP terms, and 1241 genes with 186 CC terms) and mouse species (572 genes with 149 MF terms, 880 genes with 1230 BP terms, and 737 genes with 128 CC terms). Moreover, we further compared our methods with GeneNetwork in human species (918 genes with 165 MF terms, 1230 genes with 522 BP terms, and 1328 genes with 182 CC terms), as shown in Figure S12C. From Figure S12, we find that the proposed TNP and TripletGO show the significantly better performance than GENETICA and GeneNetwork for each GO aspect. For example, from the view of Fmax, TNP achieves the improvements of 112.8%, 60.7%, and 59.1%, respectively, for MF, BP, and CC aspects of human species in comparison with GENETICA. It cannot escape our notice that the Fmax and AUPRC values of TNP and TripletGO in Figure S12 are significantly lower than the corresponding values in the previous figures (Figures S2, S8, and S9), especially for CC aspect. The reason can be explained as follows. Frist, we remove a part of genes and terms, which are not included in the GENETICA’s dataset, from our test dataset. Second, the GO annotations of genes for our work and GENETICA are originated from different databases. Specifically, the GO annotation of genes in our work are downloaded from NCBI with the version of “2021-02-23”; in GENETICA, the GO annotations are extracted from Broad Institute Molecular Signatures Database v6.2. Therefore, the GO annotations for a part of test genes are different between our work and GENETICA. For example, for gene *ARHGAP1* (Entrez ID: 392), we listed the corresponding GO annotations for CC aspect in different works as follows.

(1) Our work: GO:0005768, GO:0110165, GO:0016020, GO:0043227, GO:0043226, GO:0097708, GO:0005737, GO:0031982, GO:0097443, GO:0031410, GO:0010008, GO:0098588, GO:0031090, GO:0048471, GO:0005829 (15 terms).

(2) GENETICA: GO:0048471, GO:0110165 (2 terms).

(3) GeneNetwork: GO:0005737, GO:0016020, GO:0043230, GO:0070062, GO:0005829, GO:0098588, GO:0010008, GO:0031090, GO:0048471, GO:1903561, GO:0043227, GO:0043226, GO:0110165, GO:0031982 (14 terms).

(4) Common terms between our work and GENETICA: GO:0048471, GO:0110165 (2 terms).

(5) Common terms between our work and GeneNetwork: GO:0005737, GO:0031090, GO:0005829, GO:0016020, GO:0031982, GO:0043226, GO:0048471, GO:0043227, GO:0098588, GO:0110165, GO:0010008 (11 terms).

We can notice that there are only 2 common GO terms (GO:0048471 and GO:0110165) for the annotation of gene *ARHGAP1* between our work and GENETICA. To compare our method and GENETICA in fairness, these 2 common GO terms are used as “gold standard” for GO annotation of gene *ARHGAP1* and the remaining terms are ignored. Therefore, some predicted terms, which are considered as true positives in previous Figures (Figures S2, S8, and S9), are viewed as false positives in the comparison with GENETICA, which further leads the significant performance degradation of TNP and TripletGO in this section.
