# Supplementary material for: TripletGO: Integrating Transcript Expression Profiles with Protein Homology Inferences for Gene Function Prediction
Source: Genomics Proteomics Bioinformatics. 2022 May 11;20(5):1013–27. doi: 10.1016/j.gpb.2022.03.001 (PMC10025770; doi:10.1016/j.gpb.2022.03.001)
Supplement: Supplementary File S8 — The performances of six EPGP methods for each individual species on CAFA3 test dataset. [file mmc8.docx]

**File S8 The performances of six expression profile-based GO prediction methods for each individual species on the third CAFA challenge (CAFA3) test dataset**

We further benchmarked the performances of six expression profile-based GO prediction methods for each of 7 species on CAFA3 test dataset. Figure S13 shows the values of Fmax and AUPRC for 7 species via six expression profile-based methods. Table S12 summarizes the *P* values of Fmax and AUPRC values between TNP and other five methods in Student’s *t*-test [1] for 7 species. We can observe that TNP achieves the highest values of Fmax and AUPRC among six methods for each GO aspect in most of 7 species. Taking human species as an example, TNP achieves 11.9% and 7.8% average increases on three GO aspects for Fmax and AUPRC, respectively, in comparison with the second-best performer, *i.e.,* MR.

**Reference**

[1] Ruxton GD. The unequal variance t-test is an underused alternative to Student’s t-test and the Mann–Whitney U test. Behav Ecol 2006;17:688–90.
