# Supplementary material for: TripletGO: Integrating Transcript Expression Profiles with Protein Homology Inferences for Gene Function Prediction
Source: Genomics Proteomics Bioinformatics. 2022 May 11;20(5):1013–27. doi: 10.1016/j.gpb.2022.03.001 (PMC10025770; doi:10.1016/j.gpb.2022.03.001)
Supplement: Supplementary data 9 [file mmc9.docx]

**Table S1 The numbers of genes with GO annotation of three aspects for 20 species**

| **Database** | **Species** | **Version** | **Gene number** | **Sample number** | **GO number** | **MF number** | **BP number** | **CC number** |
| --- | --- | --- | --- | --- | --- | --- | --- | --- |
| COXPRESdb | Nematoda | Cel-m.c4-0 | 17,256 | 1780 | 3154 | 1254 | 2705 | 2018 |
|  | Dog | Cfa-m.c3-0 | 16,214 | 777 | 96 | 31 | 54 | 79 |
|  | Fly | Dme-m.c4-0 | 12,626 | 4209 | 5317 | 2729 | 4874 | 3495 |
|  | Zebrafish | Dre-m.c4-0 | 10,112 | 1423 | 2477 | 541 | 2324 | 414 |
|  | Chicken | Gga-m.c4-0 | 13,757 | 1502 | 502 | 215 | 383 | 337 |
|  | Human | Hsa-m2.c3-0 | 20,199 | 27,655 | 14,706 | 9281 | 12,362 | 13,278 |
|  | Monkey | Mcc-m.c3-0 | 15,782 | 1006 | 0 | 0 | 0 | 0 |
|  | Mouse | Mmu-m.c4-0 | 20,962 | 42,916 | 10,564 | 5646 | 8909 | 7621 |
|  | Rat | Rno-m.c4-0 | 13,751 | 42,752 | 5409 | 3594 | 4387 | 4135 |
|  | Budding yeast | Sce-m.c3-0 | 4461 | 3593 | 4107 | 3130 | 3934 | 3402 |
|  | Fission yeast | Spo-m.c3-0 | 4881 | 166 | 2743 | 1303 | 2339 | 1877 |
| ATTED-II | Arabidopsis | Ath-m.c8-0 | 20,819 | 12,686 | 11,602 | 5090 | 7927 | 8656 |
|  | Field mustard | Bra-r.c3-0 | 26,339 | 164 | 0 | 0 | 0 | 0 |
|  | Soybean | Gma-m.c4-0 | 15,746 | 1022 | 0 | 0 | 0 | 0 |
|  | Medicago | Mtr-m.c4-1 | 20,376 | 780 | 0 | 0 | 0 | 0 |
|  | Rice | Osa-m.c7-0 | 19,867 | 1775 | 82 | 59 | 69 | 55 |
|  | Poplar | Ppo-m.c3-0 | 21,910 | 557 | 0 | 0 | 0 | 0 |
|  | Tomato | Sly-m.c4-0 | 5721 | 392 | 0 | 0 | 0 | 0 |
|  | Grape | Vvi-m.c4-0 | 9421 | 258 | 0 | 0 | 0 | 0 |
|  | Maize | Zma-m.c4-0 | 10,777 | 606 | 0 | 0 | 0 | 0 |

*Note*: Gene number means the total number of genes in a species; Sample number means the number of experimental samples in microarray technology; GO number means the number of genes with GO annotation in a species; MF/BP/CC number means the number of genes with MF/BP/CC GO annotation in a species. MF, molecular function; BP, biological process; CC, cellular component.
