# Supplementary material for: TripletGO: Integrating Transcript Expression Profiles with Protein Homology Inferences for Gene Function Prediction
Source: Genomics Proteomics Bioinformatics. 2022 May 11;20(5):1013–27. doi: 10.1016/j.gpb.2022.03.001 (PMC10025770; doi:10.1016/j.gpb.2022.03.001)
Supplement: Supplementary data 10 [file mmc10.docx]

**Table S2 The details of 8 benchmark datasets constructed in our work**

| **Species** | **NTR** | **NEV** | **NTE** | **NGT_MF** | **NGT_BP** | **NGT_CC** |
| --- | --- | --- | --- | --- | --- | --- |
| Human | 12,501 | 735 | 1470 | 3841 | 11,674 | 1505 |
| Mouse | 8965 | 527 | 1054 | 2735 | 12,035 | 1188 |
| Arabidopsis | 9862 | 580 | 1160 | 2245 | 4787 | 563 |
| Rat | 4599 | 270 | 540 | 2542 | 7917 | 954 |
| Fly | 4521 | 265 | 531 | 1783 | 6123 | 857 |
| Budding Yeast | 3492 | 205 | 410 | 2025 | 4525 | 899 |
| Fission Yeast | 2332 | 137 | 274 | 1426 | 3885 | 720 |
| Nematoda | 2682 | 157 | 315 | 1160 | 4042 | 539 |

*Note*: NTR, the number of genes in training datasets; NEV, the number of genes in validation datasets; NTE, the number of genes in test datasets; NGT_MF, the total number of MF terms in training, validation and test datasets; NGT_BP, the total number of BP terms in training, validation and test datasets; NGT_CC, the total number of CC terms in training, validation and test datasets.
