# Supplementary material for: TripletGO: Integrating Transcript Expression Profiles with Protein Homology Inferences for Gene Function Prediction
Source: Genomics Proteomics Bioinformatics. 2022 May 11;20(5):1013–27. doi: 10.1016/j.gpb.2022.03.001 (PMC10025770; doi:10.1016/j.gpb.2022.03.001)
Supplement: Supplementary data 11 [file mmc11.docx]

**Table S3 The values of** $\boldsymbol{\alpha}$**,** $\boldsymbol{h}$**,** $\boldsymbol{margin}$**, and** $\boldsymbol{c}_{\boldsymbol{f}}$ **on the benchmark datasets for 8 species**

| **Species** | **GO aspect** | $\boldsymbol{\alpha}$ | $\boldsymbol{h}$ | $\boldsymbol{margin}$ | $\boldsymbol{c}_{\boldsymbol{f}}$ |
| --- | --- | --- | --- | --- | --- |
| Human | MF | 5 | 1000 | 0.01 | 0.90 |
|  | BP | 5 | 1000 | 0.01 | 0.90 |
|  | CC | 10 | 1000 | 0.01 | 0.95 |
| Mouse | MF | 5 | 1000 | 0.01 | 0.90 |
|  | BP | 3 | 1000 | 0.01 | 0.90 |
|  | CC | 2 | 1000 | 0.01 | 0.95 |
| Arabidopsis | MF | 5 | 1000 | 0.01 | 0.90 |
|  | BP | 5 | 1000 | 0.01 | 0.90 |
|  | CC | 5 | 1000 | 0.01 | 0.95 |
| Rat | MF | 3 | 1000 | 0.01 | 0.90 |
|  | BP | 5 | 1000 | 0.01 | 0.90 |
|  | CC | 5 | 1000 | 0.01 | 0.95 |
| Fly | MF | 3 | 1000 | 0.01 | 0.90 |
|  | BP | 5 | 1000 | 0.01 | 0.90 |
|  | CC | 5 | 1000 | 0.01 | 0.95 |
| Budding Yeast | MF | 3 | 1000 | 0.01 | 0.90 |
|  | BP | 5 | 1000 | 0.01 | 0.90 |
|  | CC | 5 | 1000 | 0.01 | 0.95 |
| Fission Yeast | MF | 3 | - | 0.01 | 0.90 |
|  | BP | 5 | - | 0.01 | 0.90 |
|  | CC | 5 | - | 0.01 | 0.95 |
| Nematoda | MF | 5 | 1000 | 0.01 | 0.90 |
|  | BP | 5 | 1000 | 0.01 | 0.90 |
|  | CC | 5 | 1000 | 0.01 | 0.95 |

*Note*: ‘-’ means that the PCA is not executed in the corresponding species. PCA, principal component analysis.
