# Supplementary material for: TripletGO: Integrating Transcript Expression Profiles with Protein Homology Inferences for Gene Function Prediction
Source: Genomics Proteomics Bioinformatics. 2022 May 11;20(5):1013–27. doi: 10.1016/j.gpb.2022.03.001 (PMC10025770; doi:10.1016/j.gpb.2022.03.001)
Supplement: Supplementary data 12 [file mmc12.docx]

**Table S4 The *P* values between TNP and other five expression profile-based methods for WAFmax and WAAUPRC**

| **Measure** | **GO aspect** | **(TNP, MR)** | **(TNP, PCC)** | **(TNP, MLC)** | **(TNP, SRC)** | **(TNP, ED)** |
| --- | --- | --- | --- | --- | --- | --- |
| WAFmax | MF | 8.60×10^-08^ | 1.70×10^-10^ | 6.56×10^-09^ | 3.19×10^-09^ | 8.25×10^-11^ |
|  | BP | 6.11×10^-11^ | 1.33×10^-13^ | 1.71×10^-12^ | 5.65×10^-13^ | 8.64×10^-14^ |
|  | CC | 9.24×10^-09^ | 3.67×10^-11^ | 1.21×10^-09^ | 3.85×10^-10^ | 5.60×10^-11^ |
| WAAUPRC | MF | 5.13×10^-10^ | 1.39×10^-14^ | 1.14×10^-11^ | 3.25×10^-13^ | 1.04×10^-14^ |
|  | BP | 3.04×10^-09^ | 4.43×10^-13^ | 7.37×10^-12^ | 2.61×10^-12^ | 6.69×10^-13^ |
|  | CC | 1.82×10^-13^ | 3.54×10^-15^ | 1.00×10^-13^ | 1.00×10^-13^ | 6.58×10^-15^ |

*Note*: WAFmax, weighted average maximum F1-score; WAAUPRC, weighted average area under the precision-recall curve; MR, mutual rank; PCC, Pearson correlation coefficient; MLC, metric learning for co-expression; SRC, Spearman rank correlation; ED, Euclidean distance.
