# Supplementary material for: TripletGO: Integrating Transcript Expression Profiles with Protein Homology Inferences for Gene Function Prediction
Source: Genomics Proteomics Bioinformatics. 2022 May 11;20(5):1013–27. doi: 10.1016/j.gpb.2022.03.001 (PMC10025770; doi:10.1016/j.gpb.2022.03.001)
Supplement: Supplementary data 13 [file mmc13.docx]

**Table S5 The *P* values between TNP and other five expression profile-based methods for Fmax and AUPRC on 8 species**

| **Species** | **Measure** | **GO aspect** | **(TNP, MR)** | **(TNP, PCC)** | **(TNP, MLC)** | **(TNP, SRC)** | **(TNP, ED)** |
| --- | --- | --- | --- | --- | --- | --- | --- |
| Human | Fmax | MF | 1.29×10^-04^ | 3.08×10^-08^ | 2.09×10^-12^ | 1.80×10^-05^ | 7.39×10^-08^ |
|  |  | BP | 7.83×10^-09^ | 2.32×10^-11^ | 7.00×10^-18^ | 2.00×10^-10^ | 3.23×10^-11^ |
|  |  | CC | 5.07×10^-07^ | 1.95×10^-10^ | 8.01×10^-09^ | 2.53×10^-09^ | 4.75×10^-10^ |
|  | AUPRC | MF | 2.42×10^-07^ | 3.72×10^-11^ | 1.87×10^-16^ | 7.15×10^-09^ | 2.95×10^-11^ |
|  |  | BP | 1.42×10^-07^ | 1.51×10^-11^ | 1.11×10^-16^ | 6.09×10^-10^ | 3.94×10^-11^ |
|  |  | CC | 4.64×10^-12^ | 4.30×10^-13^ | 5.30×10^-17^ | 4.64×10^-12^ | 1.52×10^-12^ |
| Mouse | Fmax | MF | 2.58×10^-04^ | 4.38×10^-07^ | 1.89×10^-10^ | 4.09×10^-03^ | 4.00×10^-09^ |
|  |  | BP | 1.24×10^-03^ | 3.46×10^-09^ | 1.16×10^-13^ | 1.48×10^-04^ | 4.55×10^-09^ |
|  |  | CC | 2.96×10^-05^ | 5.33×10^-07^ | 1.22×10^-12^ | 4.56×10^-06^ | 3.73×10^-07^ |
|  | AUPRC | MF | 2.01×10^-06^ | 1.51×10^-10^ | 2.07×10^-14^ | 4.76×10^-06^ | 4.98×10^-11^ |
|  |  | BP | 7.61×10^-04^ | 5.81×10^-09^ | 1.48×10^-14^ | 4.25×10^-06^ | 5.81×10^-09^ |
|  |  | CC | 1.65×10^-04^ | 2.14×10^-08^ | 5.98×10^-15^ | 1.65×10^-04^ | 2.91×10^-09^ |
| Arabidopsis | Fmax | MF | 1.74×10^-08^ | 5.57×10^-10^ | 3.27×10^-16^ | 1.76×10^-09^ | 1.17×10^-09^ |
|  |  | BP | 2.91×10^-07^ | 2.17×10^-09^ | 7.00×10^-12^ | 1.27×10^-07^ | 6.07×10^-10^ |
|  |  | CC | 7.50×10^-09^ | 2.30×10^-10^ | 1.07×10^-09^ | 2.27×10^-06^ | 5.81×10^-10^ |
|  | AUPRC | MF | 2.89×10^-07^ | 3.47×10^-11^ | 2.84×10^-14^ | 1.73×10^-07^ | 7.94×10^-11^ |
|  |  | BP | 1.76×10^-06^ | 6.41×10^-10^ | 4.53×10^-13^ | 1.37×10^-07^ | 1.22×10^-09^ |
|  |  | CC | 1.82×10^-11^ | 1.39×10^-12^ | 3.84×10^-15^ | 6.67×10^-10^ | 5.29×10^-12^ |
| Rat | Fmax | MF | 3.04×10^-01^ | 6.57×10^-04^ | 9.63×10^-07^ | 1.87×10^-01^ | 1.43×10^-04^ |
|  |  | BP | 1.49×10^-03^ | 4.76×10^-05^ | 1.31×10^-08^ | 1.02×10^-02^ | 1.09×10^-05^ |
|  |  | CC | 2.04×10^-01^ | 2.30×10^-04^ | 9.75×10^-07^ | 9.68×10^-03^ | 2.01×10^-04^ |
|  | AUPRC | MF | 9.02×10^-04^ | 3.74×10^-05^ | 1.43×10^-05^ | 8.78×10^-06^ | 1.63×10^-05^ |
|  |  | BP | 1.40×10^-02^ | 3.60×10^-06^ | 4.04×10^-10^ | 1.46×10^-01^ | 1.30×10^-05^ |
|  |  | CC | 9.00×10^-07^ | 1.44×10^-10^ | 1.17×10^-08^ | 1.38×10^-07^ | 6.65×10^-11^ |
| Fly | Fmax | MF | 6.69×10^-03^ | 5.35×10^-06^ | 9.31×10^-11^ | 1.02×10^-07^ | 3.97×10^-07^ |
|  |  | BP | 7.87×10^-06^ | 6.97×10^-09^ | 5.39×10^-12^ | 4.91×10^-10^ | 1.97×10^-09^ |
|  |  | CC | 3.01×10^-06^ | 8.38×10^-07^ | 2.77×10^-14^ | 1.35×10^-10^ | 5.11×10^-07^ |
|  | AUPRC | MF | 3.31×10^-01^ | 1.55×10^-05^ | 9.90×10^-14^ | 6.06×10^-10^ | 6.78×10^-06^ |
|  |  | BP | 4.03×10^-06^ | 6.66×10^-10^ | 4.77×10^-13^ | 1.34×10^-11^ | 3.24×10^-10^ |
|  |  | CC | 2.04×10^-08^ | 2.56×10^-11^ | 7.29×10^-15^ | 3.58×10^-13^ | 3.63×10^-12^ |
| Budding  Yeast | Fmax | MF | 4.17×10^-06^ | 1.05×10^-08^ | 5.22×10^-13^ | 1.05×10^-08^ | 1.22×10^-08^ |
|  |  | BP | 4.32×10^-06^ | 7.34×10^-08^ | 1.20×10^-13^ | 2.07×10^-08^ | 1.41×10^-08^ |
|  |  | CC | 3.80×10^-05^ | 4.07×10^-08^ | 1.41×10^-11^ | 1.65×10^-06^ | 2.72×10^-08^ |
|  | AUPRC | MF | 9.24×10^-06^ | 2.72×10^-09^ | 2.75×10^-15^ | 1.03×10^-10^ | 2.13×10^-09^ |
|  |  | BP | 2.98×10^-08^ | 6.08×10^-11^ | 2.64×10^-15^ | 4.84×10^-12^ | 7.64×10^-11^ |
|  |  | CC | 8.16×10^-05^ | 4.98×10^-08^ | 8.94×10^-12^ | 1.13×10^-08^ | 2.91×10^-07^ |
| Fission Yeast | Fmax | MF | 1.47×10^-05^ | 2.70×10^-02^ | 4.26×10^-07^ | 8.92×10^-04^ | 2.70×10^-02^ |
|  |  | BP | 1.05×10^-06^ | 4.87×10^-06^ | 6.31×10^-13^ | 4.48×10^-08^ | 4.87×10^-06^ |
|  |  | CC | 9.97×10^-01^ | 3.26×10^-01^ | 8.52×10^-04^ | 3.50×10^-04^ | 3.26×10^-01^ |
|  | AUPRC | MF | 6.16×10^-03^ | 8.69×10^-01^ | 3.91×10^-08^ | 8.36×10^-04^ | 8.69×10^-01^ |
|  |  | BP | 1.99×10^-06^ | 6.24×10^-06^ | 4.60×10^-12^ | 6.24×10^-06^ | 6.24×10^-06^ |
|  |  | CC | 2.41×10^-03^ | 2.63×10^-05^ | 4.24×10^-07^ | 3.51×10^-06^ | 2.63×10^-05^ |
| Nematoda | Fmax | MF | 5.71×10^-04^ | 5.20×10^-03^ | 3.19×10^-08^ | 2.68×10^-06^ | 9.79×10^-04^ |
|  |  | BP | 2.12×10^-06^ | 9.91×10^-07^ | 1.09×10^-10^ | 4.80×10^-09^ | 1.58×10^-07^ |
|  |  | CC | 1.45×10^-02^ | 1.22×10^-02^ | 3.95×10^-10^ | 6.02×10^-07^ | 2.68×10^-03^ |
|  | AUPRC | MF | 5.09×10^-04^ | 8.31×10^-05^ | 1.83×10^-11^ | 2.19×10^-08^ | 1.10×10^-05^ |
|  |  | BP | 2.09×10^-06^ | 6.80×10^-09^ | 2.96×10^-13^ | 5.79×10^-12^ | 3.42×10^-09^ |
|  |  | CC | 3.90×10^-05^ | 2.28×10^-07^ | 6.39×10^-12^ | 3.74×10^-08^ | 2.28×10^-07^ |

*Note*: Fmax, maximum F1-score; AUPRC, area under the precision-recall curve.
