# Supplementary material for: TripletGO: Integrating Transcript Expression Profiles with Protein Homology Inferences for Gene Function Prediction
Source: Genomics Proteomics Bioinformatics. 2022 May 11;20(5):1013–27. doi: 10.1016/j.gpb.2022.03.001 (PMC10025770; doi:10.1016/j.gpb.2022.03.001)
Supplement: Supplementary data 14 [file mmc14.docx]

**Table S6 The Fmax values of TNP and NON-PCA-TNP on the test dataset of human species for different sampling ratios in expression data**

| **Method** | **GO aspect** | **10%** | **30%** | **50%** | **70%** | **100%** |
| --- | --- | --- | --- | --- | --- | --- |
| TNP | MF | 0.293 | 0.307 | 0.311 | 0.307 | 0.311 |
|  | BP | 0.388 | 0.391 | 0.393 | 0.392 | 0.397 |
|  | CC | 0.565 | 0.573 | 0.573 | 0.574 | 0.577 |
| NON-PCA-TNP | MF | 0.287 | 0.291 | 0.292 | 0.294 | 0.290 |
|  | BP | 0.382 | 0.384 | 0.385 | 0.386 | 0.385 |
|  | CC | 0.564 | 0.571 | 0.572 | 0.572 | 0.573 |

*Note*: NON-PCA-TNP, triplet network-based pipeline without principal component analysis.
