# Supplementary material for: TripletGO: Integrating Transcript Expression Profiles with Protein Homology Inferences for Gene Function Prediction
Source: Genomics Proteomics Bioinformatics. 2022 May 11;20(5):1013–27. doi: 10.1016/j.gpb.2022.03.001 (PMC10025770; doi:10.1016/j.gpb.2022.03.001)
Supplement: Supplementary data 15 [file mmc15.docx]

**Table S7 The *P* values between EGPN and other eight GO prediction methods for WAFmax and WAAUPRC on the test datasets of 8 species**

| **Measure** | **GO aspect** | **(EGPN, EPGP)** | **(EGPN, GSAGP)** | **(EGPN, PSAGP)** | **(EGPN, NGP)** | **(EGPN, GPN)** | **(EGPN, EPN)** | **(EGPN, EGN)** | **(EGPN, EGP)** |
| --- | --- | --- | --- | --- | --- | --- | --- | --- | --- |
| WAFmax | MF | 2.71×10^-21^ | 3.85×10^-18^ | 2.09×10^-14^ | 9.57×10^-23^ | 2.53×10^-10^ | 7.12×10^-13^ | 4.59×10^-17^ | 1.70×10^-04^ |
|  | BP | 3.39×10^-15^ | 4.82×10^-17^ | 2.65×10^-15^ | 1.42×10^-18^ | 9.68×10^-13^ | 6.33×10^-10^ | 4.37×10^-12^ | 4.34×10^-06^ |
|  | CC | 1.71×10^-13^ | 7.18×10^-19^ | 7.49×10^-18^ | 2.07×10^-18^ | 2.09×10^-14^ | 8.98×10^-10^ | 5.02×10^-11^ | 1.96×10^-07^ |
| WAAUPRC | MF | 3.31×10^-24^ | 3.51×10^-24^ | 1.17×10^-22^ | 1.16×10^-25^ | 3.09×10^-15^ | 3.79×10^-17^ | 2.31×10^-20^ | 3.07×10^-04^ |
|  | BP | 6.93×10^-20^ | 1.24×10^-23^ | 2.99×10^-22^ | 2.12×10^-23^ | 3.66×10^-18^ | 1.52×10^-14^ | 4.97×10^-17^ | 4.87×10^-11^ |
|  | CC | 9.69×10^-08^ | 3.81×10^-16^ | 3.32×10^-15^ | 1.10×10^-14^ | 2.70×10^-10^ | 1.79×10^-05^ | 2.73×10^-06^ | 2.77×10^-04^ |

*Note*: EGPN = EPGP + GSAGP + PSAGP + NGP; GPN = GSAGP + PSAGP + NGP; EPN = EPGP + PSAGP + NGP; EGN = EPGP + GSAGP + NGP; EGP = EPGP + GSAGP + PSAGP. EPGP, expression profile-based GO prediction.
