# Supplementary material for: TripletGO: Integrating Transcript Expression Profiles with Protein Homology Inferences for Gene Function Prediction
Source: Genomics Proteomics Bioinformatics. 2022 May 11;20(5):1013–27. doi: 10.1016/j.gpb.2022.03.001 (PMC10025770; doi:10.1016/j.gpb.2022.03.001)
Supplement: Supplementary data 16 [file mmc16.docx]

**Table S8 The *P* values between EGPN and other eight GO prediction methods for Fmax and AUPRC on 8 species**

| **Species** | **Measure** | **GO aspect** | **(EGPN, EPGP)** | **(EGPN, GSAGP)** | **(EGPN, PSAGP)** | **(EGPN, NGP)** | **(EGPN, GPN)** | **(EGPN, EPN)** | **(EGPN, EGN)** | **(EGPN, EGP)** |
| --- | --- | --- | --- | --- | --- | --- | --- | --- | --- | --- |
| Human | Fmax | MF | 4.29×10^-16^ | 4.45×10^-14^ | 4.23×10^-08^ | 1.37×10^-17^ | 1.58×10^-03^ | 1.26×10^-08^ | 1.18×10^-21^ | 2.65×10^-03^ |
|  |  | BP | 2.04×10^-15^ | 1.31×10^-17^ | 5.53×10^-15^ | 1.75×10^-18^ | 7.79×10^-20^ | 2.20×10^-11^ | 2.76×10^-19^ | 5.95×10^-11^ |
|  |  | CC | 9.71×10^-12^ | 1.57×10^-16^ | 1.58×10^-15^ | 8.73×10^-17^ | 3.91×10^-12^ | 7.89×10^-09^ | 9.87×10^-14^ | 6.36×10^-11^ |
|  | AUPRC | MF | 1.18×10^-21^ | 2.03×10^-21^ | 1.84×10^-19^ | 4.08×10^-23^ | 9.18×10^-18^ | 7.82×10^-23^ | 1.05×10^-31^ | 3.66×10^-07^ |
|  |  | BP | 2.48×10^-18^ | 8.91×10^-22^ | 2.70×10^-20^ | 1.52×10^-21^ | 1.18×10^-26^ | 1.42×10^-17^ | 8.86×10^-25^ | 1.68×10^-15^ |
|  |  | CC | 5.75×10^-04^ | 3.72×10^-12^ | 1.71×10^-11^ | 3.28×10^-11^ | 5.69×10^-08^ | 4.07×10^-03^ | 6.30×10^-03^ | 5.40×10^-01^ |
| Mouse | Fmax | MF | 5.63×10^-21^ | 3.45×10^-15^ | 8.18×10^-12^ | 3.72×10^-22^ | 1.36×10^-04^ | 2.08×10^-17^ | 3.99×10^-23^ | 1.42×10^-02^ |
|  |  | BP | 3.56×10^-16^ | 2.99×10^-16^ | 1.96×10^-16^ | 6.38×10^-19^ | 1.74×10^-12^ | 1.26×10^-12^ | 3.48×10^-16^ | 6.33×10^-06^ |
|  |  | CC | 1.06×10^-12^ | 1.69×10^-16^ | 8.19×10^-17^ | 1.79×10^-17^ | 2.70×10^-18^ | 1.56×10^-12^ | 1.98×10^-10^ | 6.37×10^-01^ |
|  | AUPRC | MF | 3.98×10^-22^ | 2.04×10^-20^ | 1.73×10^-19^ | 4.01×10^-23^ | 2.16×10^-09^ | 1.19×10^-25^ | 5.99×10^-28^ | 3.62×10^-01^ |
|  |  | BP | 2.60×10^-19^ | 9.96×10^-22^ | 1.70×10^-21^ | 2.96×10^-22^ | 3.74×10^-22^ | 1.12×10^-22^ | 2.83×10^-23^ | 3.21×10^-11^ |
|  |  | CC | 7.71×10^-06^ | 9.57×10^-14^ | 2.25×10^-13^ | 3.96×10^-13^ | 1.28×10^-11^ | 1.43×10^-03^ | 2.78×10^-03^ | 2.79×10^-01^ |
| Arabidopsis | Fmax | MF | 1.52×10^-17^ | 5.58×10^-14^ | 1.06×10^-12^ | 4.93×10^-19^ | 1.39×10^-11^ | 7.77×10^-15^ | 7.09×10^-17^ | 2.28×10^-03^ |
|  |  | BP | 3.80×10^-11^ | 1.67×10^-10^ | 5.28×10^-11^ | 1.01×10^-14^ | 7.19×10^-09^ | 1.04×10^-07^ | 1.39×10^-07^ | 8.80×10^-02^ |
|  |  | CC | 1.58×10^-07^ | 5.08×10^-14^ | 1.80×10^-14^ | 1.02×10^-12^ | 3.19×10^-13^ | 5.82×10^-08^ | 2.30×10^-03^ | 4.44×10^-02^ |
|  | AUPRC | MF | 1.07×10^-20^ | 1.21×10^-21^ | 2.38×10^-20^ | 1.78×10^-22^ | 1.15×10^-14^ | 1.19×10^-22^ | 1.41×10^-26^ | 3.21×10^-06^ |
|  |  | BP | 3.96×10^-18^ | 3.10×10^-21^ | 3.17×10^-20^ | 1.39×10^-21^ | 1.62×10^-24^ | 2.99×10^-22^ | 5.88×10^-22^ | 1.49×10^-13^ |
|  |  | CC | 7.76×10^-05^ | 3.35×10^-14^ | 2.98×10^-13^ | 1.41×10^-11^ | 1.52×10^-11^ | 3.53×10^-01^ | 2.32×10^-03^ | 1.20×10^-01^ |
| Rat | Fmax | MF | 8.51×10^-18^ | 3.25×10^-11^ | 1.73×10^-11^ | 4.62×10^-19^ | 8.20×10^-01^ | 6.32×10^-15^ | 7.91×10^-16^ | 4.98×10^-01^ |
|  |  | BP | 5.04×10^-10^ | 2.14×10^-11^ | 4.67×10^-11^ | 4.26×10^-14^ | 6.72×10^-08^ | 2.01×10^-06^ | 3.11×10^-07^ | 2.46×10^-01^ |
|  |  | CC | 2.12×10^-08^ | 3.22×10^-11^ | 6.12×10^-12^ | 2.22×10^-12^ | 1.50×10^-08^ | 1.23×10^-03^ | 1.24×10^-02^ | 8.58×10^-04^ |
|  | AUPRC | MF | 1.22×10^-22^ | 6.41×10^-21^ | 6.20×10^-20^ | 1.43×10^-23^ | 4.55×10^-09^ | 2.09×10^-28^ | 9.71×10^-27^ | 2.84×10^-09^ |
|  |  | BP | 1.03×10^-16^ | 5.61×10^-20^ | 3.12×10^-19^ | 3.76×10^-20^ | 1.05×10^-21^ | 5.15×10^-14^ | 5.67×10^-20^ | 1.52×10^-03^ |
|  |  | CC | 5.31×10^-09^ | 2.13×10^-15^ | 2.28×10^-15^ | 6.98×10^-15^ | 1.08×10^-14^ | 5.16×10^-07^ | 1.91×10^-02^ | 5.95×10^-02^ |
| Fly | Fmax | MF | 1.60×10^-18^ | 1.42×10^-15^ | 1.19×10^-12^ | 1.83×10^-20^ | 6.27×10^-12^ | 5.87×10^-12^ | 5.21×10^-19^ | 7.79×10^-06^ |
|  |  | BP | 2.13×10^-12^ | 5.73×10^-15^ | 9.23×10^-13^ | 2.09×10^-16^ | 1.57×10^-14^ | 6.39×10^-09^ | 4.25×10^-16^ | 4.74×10^-05^ |
|  |  | CC | 1.00×10^-09^ | 5.94×10^-15^ | 4.35×10^-12^ | 1.00×10^-14^ | 3.24×10^-16^ | 8.24×10^-03^ | 8.18×10^-15^ | 1.64×10^-05^ |
|  | AUPRC | MF | 3.30×10^-22^ | 2.71×10^-22^ | 3.56×10^-21^ | 4.08×10^-24^ | 6.16×10^-26^ | 5.94×10^-25^ | 5.07×10^-25^ | 9.30×10^-11^ |
|  |  | BP | 2.32×10^-15^ | 1.09×10^-19^ | 5.40×10^-18^ | 1.61×10^-19^ | 1.61×10^-25^ | 2.45×10^-10^ | 1.16×10^-19^ | 3.01×10^-12^ |
|  |  | CC | 2.59×10^-10^ | 3.70×10^-19^ | 1.35×10^-17^ | 6.36×10^-18^ | 9.80×10^-21^ | 4.82×10^-02^ | 1.16×10^-15^ | 1.02×10^-04^ |
| Budding Yeast | Fmax | MF | 3.34×10^-16^ | 4.96×10^-16^ | 1.04×10^-11^ | 4.28×10^-18^ | 5.01×10^-15^ | 5.42×10^-09^ | 3.43×10^-20^ | 8.27×10^-04^ |
|  |  | BP | 8.17×10^-10^ | 1.10×10^-14^ | 1.31×10^-11^ | 3.14×10^-14^ | 1.76×10^-14^ | 7.76×10^-01^ | 1.91×10^-11^ | 4.07×10^-02^ |
|  |  | CC | 8.42×10^-02^ | 2.97×10^-12^ | 1.29×10^-07^ | 1.34×10^-09^ | 1.41×10^-08^ | 9.25×10^-01^ | 1.59×10^-02^ | 8.00×10^-03^ |
|  | AUPRC | MF | 1.15×10^-17^ | 3.63×10^-20^ | 1.53×10^-18^ | 4.30×10^-20^ | 1.61×10^-22^ | 1.23×10^-15^ | 1.43×10^-25^ | 5.33×10^-01^ |
|  |  | BP | 6.37×10^-15^ | 4.23×10^-21^ | 4.02×10^-19^ | 8.61×10^-20^ | 2.08×10^-26^ | 9.02×10^-11^ | 1.77×10^-21^ | 1.23×10^-09^ |
|  |  | CC | 5.34×10^-17^ | 3.81×10^-25^ | 1.98×10^-23^ | 2.54×10^-23^ | 8.53×10^-22^ | 1.22×10^-05^ | 2.16×10^-23^ | 4.61×10^-14^ |
| Fission Yeast | Fmax | MF | 5.83×10^-20^ | 1.68×10^-16^ | 8.72×10^-01^ | 1.95×10^-20^ | 6.87×10^-03^ | 8.26×10^-13^ | 1.14×10^-25^ | 6.42×10^-01^ |
|  |  | BP | 3.00×10^-12^ | 2.56×10^-14^ | 2.44×10^-10^ | 3.25×10^-14^ | 1.66×10^-07^ | 2.30×10^-04^ | 3.11×10^-18^ | 1.88×10^-02^ |
|  |  | CC | 5.15×10^-13^ | 1.71×10^-16^ | 3.83×10^-13^ | 3.41×10^-15^ | 3.03×10^-12^ | 4.83×10^-05^ | 7.40×10^-16^ | 3.99×10^-11^ |
|  | AUPRC | MF | 6.64×10^-20^ | 4.96×10^-20^ | 4.11×10^-18^ | 1.21×10^-20^ | 4.03×10^-09^ | 1.86×10^-16^ | 2.11×10^-28^ | 1.63×10^-07^ |
|  |  | BP | 1.93×10^-18^ | 3.65×10^-22^ | 2.38×10^-19^ | 5.18×10^-21^ | 7.43×10^-17^ | 1.56×10^-11^ | 3.91×10^-27^ | 1.41×10^-08^ |
|  |  | CC | 5.39×10^-14^ | 1.01×10^-19^ | 9.20×10^-18^ | 2.48×10^-18^ | 9.57×10^-22^ | 3.94×10^-01^ | 1.37×10^-18^ | 5.73×10^-07^ |
| Nematoda | Fmax | MF | 3.62×10^-14^ | 6.79×10^-11^ | 8.72×10^-01^ | 8.85×10^-16^ | 8.91×10^-01^ | 7.73×10^-08^ | 6.60×10^-18^ | 7.18×10^-03^ |
|  |  | BP | 2.31×10^-08^ | 9.59×10^-12^ | 9.80×10^-10^ | 1.39×10^-12^ | 4.25×10^-12^ | 5.61×10^-03^ | 1.40×10^-09^ | 7.70×10^-01^ |
|  |  | CC | 8.47×10^-08^ | 6.57×10^-15^ | 4.39×10^-12^ | 9.90×10^-13^ | 3.53×10^-07^ | 1.69×10^-02^ | 1.04×10^-10^ | 1.90×10^-03^ |
|  | AUPRC | MF | 2.34×10^-17^ | 1.28×10^-17^ | 2.23×10^-13^ | 1.34×10^-18^ | 1.00×10^-01^ | 2.24×10^-01^ | 3.21×10^-23^ | 5.07×10^-02^ |
|  |  | BP | 6.74×10^-14^ | 5.92×10^-19^ | 4.85×10^-17^ | 3.38×10^-18^ | 3.54×10^-22^ | 6.00×10^-08^ | 1.48×10^-17^ | 5.85×10^-02^ |
|  |  | CC | 1.33×10^-09^ | 1.28×10^-18^ | 3.85×10^-16^ | 3.71×10^-16^ | 2.44×10^-15^ | 5.88×10^-01^ | 1.81×10^-10^ | 9.70×10^-02^ |
