# Supplementary material for: TripletGO: Integrating Transcript Expression Profiles with Protein Homology Inferences for Gene Function Prediction
Source: Genomics Proteomics Bioinformatics. 2022 May 11;20(5):1013–27. doi: 10.1016/j.gpb.2022.03.001 (PMC10025770; doi:10.1016/j.gpb.2022.03.001)
Supplement: Supplementary data 17 [file mmc17.docx]

**Table S9 The *P* values between EGN and other six GO prediction methods for Fmax and AUPRC on 98 non-coding genes**

| **Measure** | **GO aspect** | **(EGN, EPGP)** | **(EGN, GSAGP)** | **(EGN, NGP)** | **(EGN, GN)** | **(EGN, EN)** | **(EGN, EG)** |
| --- | --- | --- | --- | --- | --- | --- | --- |
| Fmax | MF | 1.09×10^-12^ | 4.01×10^-17^ | 5.48×10^-18^ | 4.01×10^-17^ | 1.95×10^-12^ | 4.16×10^-10^ |
|  | BP | 3.86×10^-10^ | 3.36×10^-18^ | 1.07×10^-16^ | 7.98×10^-17^ | 3.86×10^-10^ | 1.39×10^-03^ |
|  | CC | 1.73×10^-05^ | 1.35×10^-13^ | 1.35×10^-13^ | 1.17×10^-11^ | 1.80×10^-03^ | 2.80×10^-05^ |
| AUPRC | MF | 4.83×10^-20^ | 3.39×10^-25^ | 2.87×10^-25^ | 4.30×10^-24^ | 1.13×10^-19^ | 6.30×10^-17^ |
|  | BP | 5.90×10^-09^ | 1.51×10^-23^ | 1.99×10^-21^ | 9.56×10^-21^ | 1.38×10^-09^ | 5.88×10^-11^ |
|  | CC | 1.56×10^-11^ | 8.53×10^-25^ | 2.93×10^-24^ | 3.07×10^-22^ | 1.56×10^-11^ | 1.01×10^-05^ |

*Note*: GN = GSAGP + NPG; EN = EPGP + NPG; EG = EPGP + GSAGP.
