# Supplementary material for: TripletGO: Integrating Transcript Expression Profiles with Protein Homology Inferences for Gene Function Prediction
Source: Genomics Proteomics Bioinformatics. 2022 May 11;20(5):1013–27. doi: 10.1016/j.gpb.2022.03.001 (PMC10025770; doi:10.1016/j.gpb.2022.03.001)
Supplement: Supplementary data 18 [file mmc18.docx]

**Table S10 The details of training and test dataset for 7 species in CAFA3 dataset**

| **Species** | **NTR** | **NTE** | **NGT_MF** | **NGT_BP** | **NGT_CC** |
| --- | --- | --- | --- | --- | --- |
| Human | 13,639 | 1020 | 3611 | 10,680 | 1348 |
| Mouse | 9220 | 277 | 2374 | 10,949 | 1004 |
| Arabidopsis | 7550 | 470 | 1958 | 4230 | 516 |
| Rat | 3645 | 75 | 2198 | 6684 | 784 |
| Fly | 2420 | 196 | 1263 | 4875 | 693 |
| Budding Yeast | 4061 | 23 | 1986 | 4511 | 877 |
| Fission Yeast | 4237 | 372 | 1237 | 3824 | 626 |

*Note*: CAFA3, the third Critical Assessment of Protein Function Annotation.
