# Supplementary material for: TripletGO: Integrating Transcript Expression Profiles with Protein Homology Inferences for Gene Function Prediction
Source: Genomics Proteomics Bioinformatics. 2022 May 11;20(5):1013–27. doi: 10.1016/j.gpb.2022.03.001 (PMC10025770; doi:10.1016/j.gpb.2022.03.001)
Supplement: Supplementary data 19 [file mmc19.docx]

**Table S11 The *P* values between TNP and other five expression profile-based methods for Fmax and AUPRC on 2433 proteins of 7 species from CAFA3 test dataset**

| **Measure** | **GO aspect** | **(TNP, MR)** | **(TNP, PCC)** | **(TNP, MLC)** | **(TNP, SRC)** | **(TNP, ED)** |
| --- | --- | --- | --- | --- | --- | --- |
| Fmax | MF | 8.07×10^-09^ | 4.92×10^-09^ | 2.25×10^-15^ | 7.16×10^-09^ | 1.29×10^-09^ |
|  | BP | 2.87×10^-07^ | 3.95×10^-09^ | 5.38×10^-14^ | 2.07×10^-08^ | 8.28×10^-09^ |
|  | CC | 2.14×10^-07^ | 8.38×10^-09^ | 3.53×10^-12^ | 1.45×10^-07^ | 3.72×10^-09^ |
| AUPRC | MF | 1.41×10^-01^ | 3.10×10^-05^ | 2.41×10^-11^ | 3.13×10^-04^ | 1.15×10^-05^ |
|  | BP | 2.49×10^-05^ | 4.09×10^-07^ | 2.76×10^-13^ | 6.15×10^-06^ | 1.15×10^-06^ |
|  | CC | 8.01×10^-01^ | 3.59×10^-08^ | 7.11×10^-05^ | 4.46×10^-02^ | 1.04×10^-08^ |
