# Supplementary material for: TripletGO: Integrating Transcript Expression Profiles with Protein Homology Inferences for Gene Function Prediction
Source: Genomics Proteomics Bioinformatics. 2022 May 11;20(5):1013–27. doi: 10.1016/j.gpb.2022.03.001 (PMC10025770; doi:10.1016/j.gpb.2022.03.001)
Supplement: Supplementary data 20 [file mmc20.docx]

**Table S12 The *P* values between TNP and other five expression profile-based methods for Fmax and AUPRC on CAFA3 test dataset for each of 7 species**

| **Species** | **Measure** | **GO aspect** | **(TNP, MR)** | **(TNP, PCC)** | **(TNP, MLC)** | **(TNP, SRC)** | **(TNP, ED)** |
| --- | --- | --- | --- | --- | --- | --- | --- |
| Human | Fmax | MF | 1.69×10^-02^ | 4.86×10^-05^ | 2.09×10^-04^ | 4.09×10^-03^ | 9.32×10^-06^ |
|  |  | BP | 1.48×10^-02^ | 4.96×10^-06^ | 2.54×10^-09^ | 1.57×10^-03^ | 2.15×10^-05^ |
|  |  | CC | 3.58×10^-04^ | 6.87×10^-07^ | 5.46×10^-09^ | 1.09×10^-05^ | 2.79×10^-07^ |
|  | AUPRC | MF | 4.64×10^-04^ | 2.80×10^-04^ | 9.63×10^-01^ | 4.54×10^-05^ | 3.01×10^-05^ |
|  |  | BP | 7.63×10^-02^ | 3.40×10^-04^ | 3.79×10^-08^ | 1.37×10^-01^ | 3.87×10^-03^ |
|  |  | CC | 9.28×10^-02^ | 4.54×10^-08^ | 4.59×10^-03^ | 9.28×10^-04^ | 4.54×10^-08^ |
| Mouse | Fmax | MF | 6.45×10^-06^ | 3.51×10^-08^ | 8.42×10^-09^ | 3.32×10^-07^ | 1.70×10^-07^ |
|  |  | BP | 8.47×10^-08^ | 3.37×10^-08^ | 1.05×10^-11^ | 2.31×10^-07^ | 1.39×10^-07^ |
|  |  | CC | 1.98×10^-04^ | 3.74×10^-03^ | 2.39×10^-04^ | 5.25×10^-03^ | 1.23×10^-04^ |
|  | AUPRC | MF | 7.05×10^-01^ | 9.43×10^-07^ | 3.90×10^-06^ | 8.26×10^-02^ | 2.30×10^-06^ |
|  |  | BP | 3.54×10^-02^ | 1.55×10^-05^ | 8.05×10^-08^ | 2.27×10^-01^ | 2.01×10^-06^ |
|  |  | CC | 8.23×10^-09^ | 4.69×10^-07^ | 2.96×10^-10^ | 2.28×10^-08^ | 8.67×10^-07^ |
| Arabidopsis | Fmax | MF | 6.05×10^-06^ | 2.64×10^-06^ | 6.28×10^-08^ | 1.56×10^-04^ | 7.72×10^-07^ |
|  |  | BP | 1.87×10^-02^ | 6.95×10^-05^ | 6.53×10^-12^ | 1.41×10^-05^ | 1.10×10^-04^ |
|  |  | CC | 7.25×10^-05^ | 6.74×10^-07^ | 2.14×10^-05^ | 6.90×10^-06^ | 1.53×10^-07^ |
|  | AUPRC | MF | 2.48×10^-03^ | 2.48×10^-03^ | 3.16×10^-06^ | 5.81×10^-01^ | 3.41×10^-04^ |
|  |  | BP | 3.06×10^-03^ | 1.24×10^-05^ | 1.32×10^-13^ | 4.61×10^-08^ | 3.89×10^-05^ |
|  |  | CC | 6.33×10^-03^ | 1.60×10^-04^ | 4.47×10^-06^ | 3.20×10^-03^ | 2.76×10^-04^ |
| Rat | Fmax | MF | 5.23×10^-01^ | 3.58×10^-01^ | 5.07×10^-04^ | 9.21×10^-03^ | 2.41×10^-01^ |
|  |  | BP | 3.08×10^-01^ | 8.43×10^-06^ | 1.94×10^-02^ | 1.56×10^-01^ | 4.71×10^-06^ |
|  |  | CC | 2.76×10^-02^ | 4.50×10^-02^ | 3.07×10^-04^ | 6.25×10^-01^ | 1.93×10^-02^ |
|  | AUPRC | MF | 4.14×10^-01^ | 4.14×10^-01^ | 3.48×10^-03^ | 9.47×10^-02^ | 1.37×10^-01^ |
|  |  | BP | 1.12×10^-04^ | 2.88×10^-02^ | 5.56×10^-04^ | 1.32×10^-06^ | 1.90×10^-03^ |
|  |  | CC | 6.57×10^-03^ | 1.87×10^-02^ | 2.39×10^-03^ | 9.17×10^-02^ | 6.57×10^-03^ |
| Fly | Fmax | MF | 6.79×10^-03^ | 1.08×10^-01^ | 5.13×10^-05^ | 8.70×10^-04^ | 1.23×10^-02^ |
|  |  | BP | 3.62×10^-03^ | 5.03×10^-02^ | 3.94×10^-07^ | 8.67×10^-04^ | 2.66×10^-03^ |
|  |  | CC | 2.40×10^-04^ | 1.48×10^-04^ | 1.36×10^-06^ | 2.42×10^-05^ | 4.67×10^-06^ |
|  | AUPRC | MF | 5.84×10^-06^ | 1.03×10^-05^ | 8.06×10^-11^ | 1.48×10^-08^ | 2.05×10^-06^ |
|  |  | BP | 1.16×10^-03^ | 2.01×10^-04^ | 5.38×10^-10^ | 1.21×10^-08^ | 8.70×10^-03^ |
|  |  | CC | 3.51×10^-04^ | 6.82×10^-06^ | 1.02×10^-06^ | 1.20×10^-05^ | 6.14×10^-07^ |
| Budding  Yeast | Fmax | MF | 2.63×10^-04^ | 8.95×10^-02^ | 5.03×10^-04^ | 3.36×10^-06^ | 1.90×10^-03^ |
|  |  | BP | 1.02×10^-01^ | 1.28×10^-06^ | 1.28×10^-02^ | 2.08×10^-05^ | 2.99×10^-06^ |
|  |  | CC | 2.39×10^-03^ | 1.17×10^-02^ | 2.89×10^-04^ | 1.67×10^-03^ | 1.17×10^-02^ |
|  | AUPRC | MF | 8.79×10^-01^ | 4.29×10^-02^ | 9.93×10^-06^ | 4.20×10^-08^ | 4.29×10^-02^ |
|  |  | BP | 6.74×10^-01^ | 6.74×10^-01^ | 6.41×10^-01^ | 4.03×10^-04^ | 7.47×10^-01^ |
|  |  | CC | 2.55×10^-05^ | 9.48×10^-03^ | 6.52×10^-01^ | 1.17×10^-05^ | 2.74×10^-03^ |
| Fission Yeast | Fmax | MF | 7.90×10^-01^ | 5.46×10^-01^ | 6.95×10^-03^ | 3.88×10^-01^ | 7.58×10^-01^ |
|  |  | BP | 2.72×10^-10^ | 7.08×10^-11^ | 2.30×10^-16^ | 3.86×10^-11^ | 7.08×10^-11^ |
|  |  | CC | 3.42×10^-02^ | 4.53×10^-05^ | 4.31×10^-05^ | 1.38×10^-04^ | 4.53×10^-05^ |
|  | AUPRC | MF | 9.89×10^-06^ | 3.37×10^-06^ | 7.07×10^-09^ | 3.31×10^-05^ | 3.37×10^-06^ |
|  |  | BP | 7.06×10^-13^ | 3.08×10^-13^ | 1.59×10^-19^ | 2.49×10^-13^ | 3.08×10^-13^ |
|  |  | CC | 3.37×10^-04^ | 9.72×10^-03^ | 6.07×10^-03^ | 4.84×10^-03^ | 8.15×10^-03^ |
