# Supplementary material for: TripletGO: Integrating Transcript Expression Profiles with Protein Homology Inferences for Gene Function Prediction
Source: Genomics Proteomics Bioinformatics. 2022 May 11;20(5):1013–27. doi: 10.1016/j.gpb.2022.03.001 (PMC10025770; doi:10.1016/j.gpb.2022.03.001)
Supplement: Supplementary data 21 [file mmc21.docx]

**Table S13 The *P* values between TripletGO and other six GO prediction methods for Fmax and AUPRC on 2433 proteins of 7 species from CAFA3 test dataset**

| **Measure** | **GO aspect** | **(TripletGO, EPGP)** | **(TripletGO, GSAGP)** | **(TripletGO, PSAGP)** | **(TripletGO, NGP)** | **(TripletGO, DeepGO)** | **(TripletGO, FunFams)** |
| --- | --- | --- | --- | --- | --- | --- | --- |
| Fmax | MF | 4.75×10^-19^ | 4.07×10^-16^ | 9.05×10^-02^ | 8.09×10^-20^ | 8.74×10^-18^ | 2.07×10^-07^ |
|  | BP | 2.79×10^-08^ | 1.42×10^-11^ | 5.90×10^-07^ | 2.12×10^-13^ | 5.44×10^-09^ | 1.83×10^-07^ |
|  | CC | 5.99×10^-10^ | 1.56×10^-14^ | 9.02×10^-10^ | 1.31×10^-12^ | 1.63×10^-10^ | 7.58×10^-14^ |
| AUPRC | MF | 5.58×10^-22^ | 1.22×10^-21^ | 3.60×10^-19^ | 1.36×10^-22^ | 1.88×10^-20^ | 1.69×10^-18^ |
|  | BP | 2.12×10^-15^ | 1.12×10^-18^ | 1.93×10^-14^ | 2.21×10^-18^ | 4.69×10^-16^ | 1.37×10^-17^ |
|  | CC | 2.09×10^-11^ | 2.11×10^-19^ | 5.95×10^-17^ | 2.38×10^-16^ | 1.46×10^-11^ | 1.62×10^-18^ |
