# Supplementary material for: TripletGO: Integrating Transcript Expression Profiles with Protein Homology Inferences for Gene Function Prediction
Source: Genomics Proteomics Bioinformatics. 2022 May 11;20(5):1013–27. doi: 10.1016/j.gpb.2022.03.001 (PMC10025770; doi:10.1016/j.gpb.2022.03.001)
Supplement: Supplementary data 22 [file mmc22.docx]

**Table S14 The confidence scores of the candidate GO terms for gene *GALNT4* by TripletGO**

| **GO terms** | **GO aspect** | **Confidence score** |  |  |  | **GO terms** | **GO aspect** | **Confidence score** |
| --- | --- | --- | --- | --- | --- | --- | --- | --- |
| **GO:0110165** | CC | 0.968 |  |  |  | **GO:0016020** | CC | 0.771 |
| **GO:0031090** | CC | 0.700 |  |  |  | **GO:0043226** | CC | 0.626 |
| **GO:0043227** | CC | 0.602 |  |  |  | **GO:0098588** | CC | 0.534 |
| **GO:0005622** | CC | 0.417 |  |  |  | **GO:0043229** | CC | 0.415 |
| **GO:0000139** | CC | 0.405 |  |  |  | **GO:0043231** | CC | 0.369 |
| GO:0031982 | CC | 0.325 |  |  |  | GO:0097708 | CC | 0.284 |
| GO:0031410 | CC | 0.284 |  |  |  | GO:0005886 | CC | 0.265 |
| GO:0005829 | CC | 0.184 |  |  |  | GO:0005789 | CC | 0.177 |
| GO:0030133 | CC | 0.166 |  |  |  | GO:0005654 | CC | 0.141 |
| GO:0005737 | CC | 0.136 |  |  |  | GO:0005783 | CC | 0.135 |
| GO:0031224 | CC | 0.134 |  |  |  | GO:0016021 | CC | 0.134 |
| GO:0005794 | CC | 0.112 |  |  |  | GO:0032991 | CC | 0.087 |
| GO:0031226 | CC | 0.084 |  |  |  | GO:0005887 | CC | 0.084 |
| GO:0005634 | CC | 0.070 |  |  |  | GO:0043228 | CC | 0.049 |
| GO:0043232 | CC | 0.047 |  |  |  | GO:0098590 | CC | 0.045 |
| GO:0098796 | CC | 0.043 |  |  |  | GO:0031974 | CC | 0.037 |
| GO:0012506 | CC | 0.037 |  |  |  | GO:0042995 | CC | 0.036 |
| GO:0120025 | CC | 0.036 |  |  |  | GO:0043233 | CC | 0.035 |
| GO:0030659 | CC | 0.034 |  |  |  | GO:0005768 | CC | 0.031 |
| GO:0070013 | CC | 0.027 |  |  |  | GO:0030054 | CC | 0.024 |
| GO:0005739 | CC | 0.022 |  |  |  | GO:0031301 | CC | 0.022 |
| GO:0031300 | CC | 0.022 |  |  |  | GO:0010008 | CC | 0.021 |
| GO:1902494 | CC | 0.019 |  |  |  | GO:0048471 | CC | 0.019 |
| GO:0030667 | CC | 0.018 |  |  |  | GO:0016604 | CC | 0.017 |
| GO:0005815 | CC | 0.015 |  |  |  | GO:0005576 | CC | 0.014 |
| GO:0005774 | CC | 0.012 |  |  |  | GO:0098852 | CC | 0.012 |
| GO:0005765 | CC | 0.012 |  |  |  | GO:1990234 | CC | 0.011 |
| GO:0070161 | CC | 0.010 |  |  |  | GO:0005929 | CC | 0.010 |

*Note*: Cut-off value = 0.350; The GO terms which are predicted as positives by TripletGO are highlighted in bold fonts.
